# A novel phosphorylation site involved in dissociating RAF kinase from the scaffolding protein 14-3-3 and disrupting RAF dimerization

**DOI:** 10.1016/j.jbc.2023.105188

**Published:** 2023-08-23

**Authors:** Alison Yu, Duc Huy Nguyen, Thomas Joseph Nguyen, Zhihong Wang

**Affiliations:** Department of Chemistry & Biochemistry, Rowan University, Glassboro, New Jersey, USA

**Keywords:** phosphorylation, RAF kinase, 14-3-3, scaffolding protein, cancer, dimerization, ATP-competitive inhibitor, paradoxical activation, regulatory mechanism, kinase signaling

## Abstract

Rapidly accelerated fibrosarcoma (ARAF, BRAF, CRAF) kinase is central to the MAPK pathway (RAS–RAF–MEK–ERK). Inactive RAF kinase is believed to be monomeric, autoinhibited, and cytosolic, while activated RAF is recruited to the membrane *via* RAS-GTP, leading to the relief of autoinhibition, phosphorylation of key regulatory sites, and dimerization of RAF protomers. Although it is well known that active and inactive BRAF have differential phosphorylation sites that play a crucial role in regulating BRAF, key details are still missing. In this study, we report the characterization of a novel phosphorylation site, BRAF^S732^ (equivalent in CRAF^S624^), located in proximity to the C-terminus binding motif for the 14-3-3 scaffolding protein. At the C terminus, 14-3-3 binds to BRAF^pS729^ (CRAF^pS621^) and enhances RAF dimerization. We conducted mutational analysis of BRAF^S732A/E^ and CRAF^S624A/E^ and revealed that the phosphomimetic S→E mutant decreases 14-3-3 association and RAF dimerization. In normal cell signaling, dimerized RAF phosphorylates MEK1/2, which is observed in the phospho-deficient S→A mutant. Our results suggest that phosphorylation and dephosphorylation of this site fine-tune the association of 14-3-3 and RAF dimerization, ultimately impacting MEK phosphorylation. We further characterized the BRAF homodimer and BRAF:CRAF heterodimer and identified a correlation between phosphorylation of this site with drug sensitivity. Our work reveals a novel negative regulatory role for phosphorylation of BRAF^S732^ and CRAF^S624^ in decreasing 14-3-3 association, dimerization, and MEK phosphorylation. These findings provide insight into the regulation of the MAPK pathway and may have implications for cancers driven by mutations in the pathway.

RAF kinases are essential components of the MAPK (RAS–RAF–MEK–ERK) pathway, which governs key cellular processes including cell growth, differentiation, proliferation, and migration (1, 2). Among the three RAF isoforms (ARAF, BRAF, and CRAF/Raf-1), BRAF is the most frequently mutated isoform in cancers. Despite the critical role of RAF in oncogenesis, the current model of RAF regulation is incomplete. The process of RAF activation involves multiple steps including phosphorylation, autoinhibitory relief, dimerization, and membrane recruitment (1). Existing therapies for oncogenic RAF are limited by paradoxical activation, secondary malignancies, and off-target effects, highlighting the need for a more comprehensive understanding of the regulatory mechanisms (3).

The three isoforms of RAF share three conserved regions (CR1/2/3). CR1 contains the RAS-binding domain and the cysteine-rich domain (CRD). The RAS-binding domain interacts with canonical activated RAS-GTP at the plasma membrane to initiate RAF activation (4). In inactive RAF, the CRD makes extensive contacts with the dimeric 14-3-3 scaffolding protein and the kinase domain, which sequesters the RAF dimer interface to prevent dimerization (5). CR2 is a serine/threonine-rich region that contains the N-terminal region phospho-serine site (BRAF^pS365^) within a RSXpSXP motif recognized by the 14-3-3 scaffolding protein; CR2 interacts with 14-3-3 to lock BRAF in its autoinhibitory conformation (6, 7). CR3) contains the well-studied kinase domain. Dimerization of the kinase domain is crucial for normal RAF activation (8). The BRAF:CRAF heterodimer has been implicated in maximum MAPK pathway output, followed by the BRAF homodimer; CRAF homodimers have very low–basal activity (9). Dimerized and active RAF phosphorylates sole substrate MEK1/2 to continue the MAPK pathway.

CR3 contains a constitutively phosphorylated C-terminal region serine (BRAF^S729^/CRAF^S621^) that is recognized by the 14-3-3 scaffolding protein. Phosphorylation of this C-terminus site has been suggested by activated protein kinase, PKA, and autophosphorylation of the activation loop (BRAF^pT599^) (10, 11). The 14-3-3 scaffolding protein exerts both a positive-activating and negative-inactivating role in RAF kinase. The autoinhibited BRAF cryo-EM structure reveals that the CRD and the kinase domain makes contacts with a 14-3-3 dimer at the N-terminal region 14-3-3 recognition site, BRAF^pS365^ (5). Although the sequential order of regulatory events remains unclear, dephosphorylation of BRAF^pS365^ releases the autoinhibitory interaction of the N-terminal region 14-3-3 scaffolding protein to promote increased RAF activity (1). At the C terminus, 14-3-3 binding is believed to facilitate dimerization of two RAF protomers, acting as a positive regulator of RAF kinase regulation (12, 13). It remains unclear how the C terminal region 14-3-3 association event is subject to regulation.

BRAF is the most frequently mutated kinase in cancer, accounting for 8% of all cancers, but attempts to target BRAF have been problematic (1). Currently, there are three U.S. Food and Drug Administration (FDA)-approved inhibitors (vemurafenib, dabrafenib, and encorafenib) that target BRAF^V600E/K^ (14, 15). However, these inhibitors can result in paradoxical activation, an upregulation of the pathway producing secondary malignancies (16). These FDA-approved inhibitors do not address the clinical needs of non-BRAF^V600^ mutants; non-BRAF^V600^ mutants encompass roughly 50% of BRAF mutants in non-small cell lung cancer patients and 30% of all cancer patients contain an upstream RAS mutation (17). To better design the next generation therapeutics, a better understanding of the regulatory mechanisms of the RAF pathway is needed.

Phosphorylation and dephosphorylation tightly regulate the pathway output of RAF kinase. In this study, we report the characterization of BRAF^S732^ and CRAF^S624^. BRAF^pS732^ was identified by affinity-captured LC-MS/MS (Tables S1 and S2, phosphorylated peptide identification). BRAF^S732^ is located posterior to the C-terminal region 14-3-3 binding site (Fig. 1). We hypothesized that phosphorylation of BRAF^S732^ would disrupt the binding interaction due to its proximity to a known 14-3-3 scaffolding protein interaction motif at the C terminus of BRAF. Using mutational analysis, phospho-deficient BRAF^S732A^ and phosphomimetic BRAF^S732D/E^, our coimmunoprecipitation (co-IP) data supports that phosphomimetic mutants decrease 14-3-3 binding at the C terminus. Additionally, we found that the phosphomimetic mutant BRAF^S732E^ displays decreased activation loop phosphorylation, whereas the phospho-deficient BRAF^S732A^ exhibits the opposite effect, promoting activation loop phosphorylation. Furthermore, the phosphomimetic BRAF^S732E^ reduces BRAF homodimerization and BRAF:CRAF heterodimerization. Concurrently, the homologous CRAF phospho-deficient^S624A^ mutant displays the similar behavior. Collectively, our data suggest that the conserved serine in BRAF^S732^ and CRAF^S6^^2^^4^ acts as a negative regulator of BRAF and CRAF, providing further complexity to a tightly regulated system.

**Figure 1:**
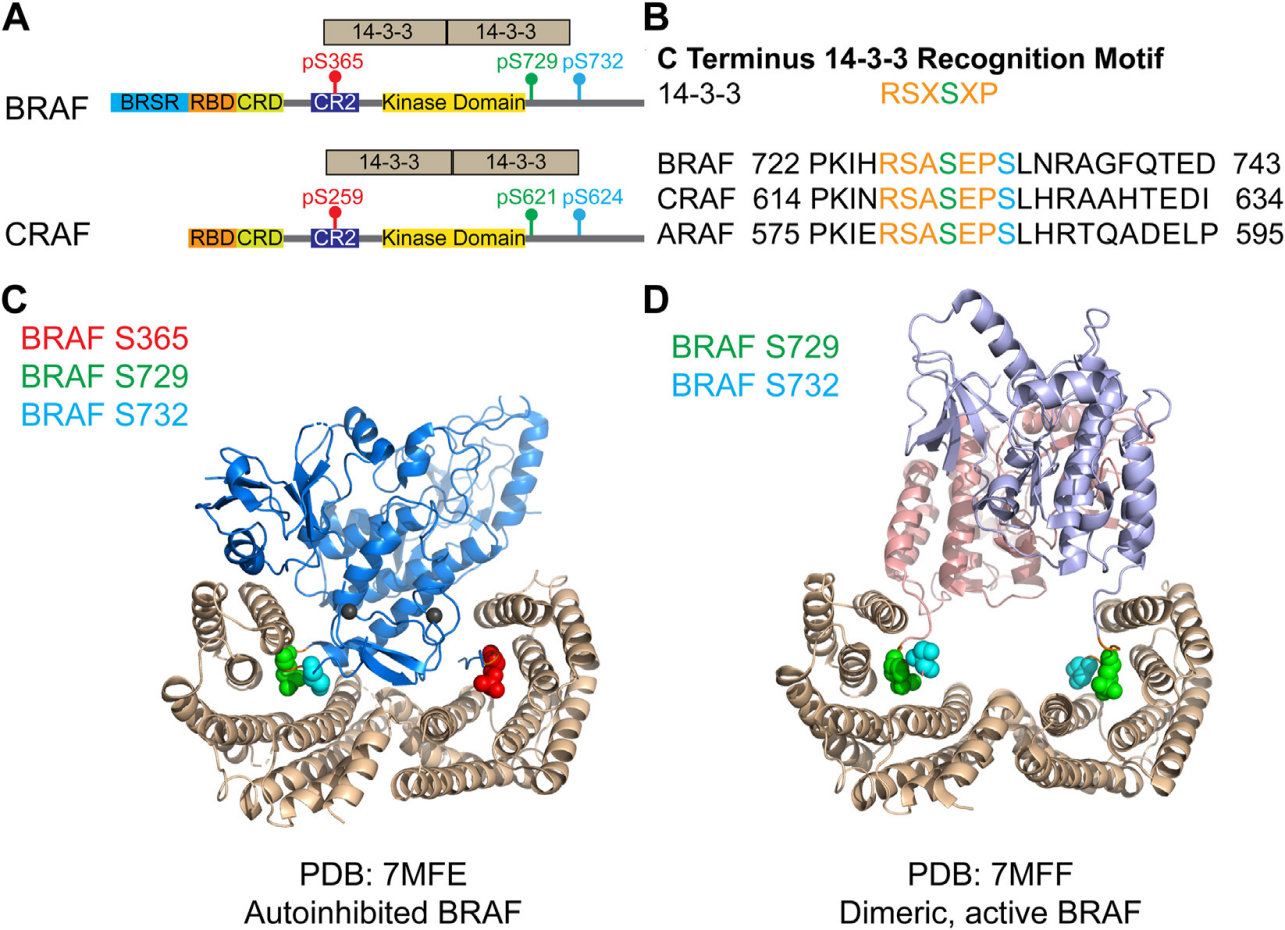
**BRAF**^**S732**^**is located in proximity to a known 14-3-3 scaffolding protein interaction region.***A*, architecture of BRAF and CRAF with respect to the dimeric 14-3-3 scaffolding protein (*beige*) phospho-serine binding sites at the N (amino) and C (carboxyl) terminus of the kinase. The RAF family proteins contain three conserved regions that are composed of the RBD (Ras-binding domain), CRD (cysteine-rich domain), conserved region 2, and the kinase domain. The BRSR is unique to BRAF, which is the BRAF-specific region. Highlighted phospho-serine sites in *red* (BRAF^S365^) and *green* (BRAF^S729^) are phospho-serines that are recognized by the 14-3-3 scaffolding protein (*beige*). *B*, mass spectrometry captured phosphorylated BRAF^S732^, which is in proximity to a known interaction region of the 14-3-3 scaffolding protein. 14-3-3 is a dimeric scaffolding protein that recognizes the RSXsXP motif of BRAF at the C-terminus region. BRAF^pS729^ (in *green* to represent the phospho-serine) site is constitutively phosphorylated. Mass spectrometry captured phosphorylated BRAF^S732^ (*cyan*). This site is conserved in all three mammalian isoforms of RAF. *C*, BRAF^S732^ (*cyan, space-filling sphere*) highlighted in the cryo-EM structures of autoinhibited BRAF (*C*, *blue*) in complex with the 14-3-3 (*beige*) scaffolding protein. Autoinhibited BRAF is phosphorylated at S365 (*red*, *space-filling sphere*) and S729 (*green, space-filling sphere*). The recognition motif is colored *orange* and recognizes the phosphorylated serine within the RSXSXP recognition motif. PDB: 7MFE. *D*, dimeric, active BRAF contains two protomers (*purple* and *pink* represent two protomers of the BRAF dimer) that are phosphorylated at S729. These two BRAF phospho-S729 enable recognition by the dimeric 14-3-3 scaffolding protein. Dimeric BRAF^S732^ is within the 14-3-3 interaction region. PDB: 7MFF.

## Results

### BRAF^pS732^ resides in proximity to a known 14-3-3 interaction region

Using an engineered membrane localization system, cytosolic BRAF-FLAG was membrane recruited and captured using immunoprecipitation. Post translational modifications of cytosolic and membrane localized BRAF-FLAG were identified using affinity-captured LC-MS/MS. In two biological replicates, BRAF^pS732^ was identified in both populations along with other reported known and characterized phosphorylation sites (Supporting information 1–2, identified phosphosites). BRAF^S732^ is preceded by the C-terminus 14-3-3 recognition motif (Fig. 1*A*, green BRAF^pS729^), and this serine is well conserved among the three human RAFs (ARAF, BRAF, and CRAF) (Fig. 1*A*, red). Phosphorylation sites were searched against available literature and databases. The PhosphoSite database reports that BRAF ^pS732^ has been previously annotated by three internal PhosphoSite database curation sets: 12,432, 9879, and 9883 (18). In addition, two clinical samples contain a missense phenylalanine mutation in the COSMIC database in carcinoma and malignant melanoma (19, 20). The conserved CRAF^pS624^ site has been previously identified (21, 22, 23). Despite identical sequence conservation and phospho-serine585, ARAF was omitted due to its low-intrinsic basal kinase activity (24). The autoinhibited and dimeric BRAF cryo-EM structures in complex with 14-3-3 dimers reveal BRAF^S732^ (cyan, space filled atoms) to be present in the 14-3-3 binding groove (beige) (Fig. 1, *B* and *C*), although BRAF^S732^ is located outside of the C-terminus BRAF^S729^ (green) 14-3-3 recognition motif (RSXpSXP) (25). C-terminus 14-3-3 dimers facilitate the dimerization of two RAF protomers (5). Phosphorylation typically influences the local environment, and the enzyme may be allosterically modified. Based on the localization of BRAF^S732^ in complex with 14-3-3 (Fig. 1), we rationalized that phosphorylation may interfere with the known 14-3-3 scaffolding protein association at the C terminus.

### BRAF^S732^ phosphomimetic mutations decrease 14-3-3 scaffolding protein association with BRAF at the C terminus, activation loop phosphorylation, and the BRAF homodimer

**Figure 2:**
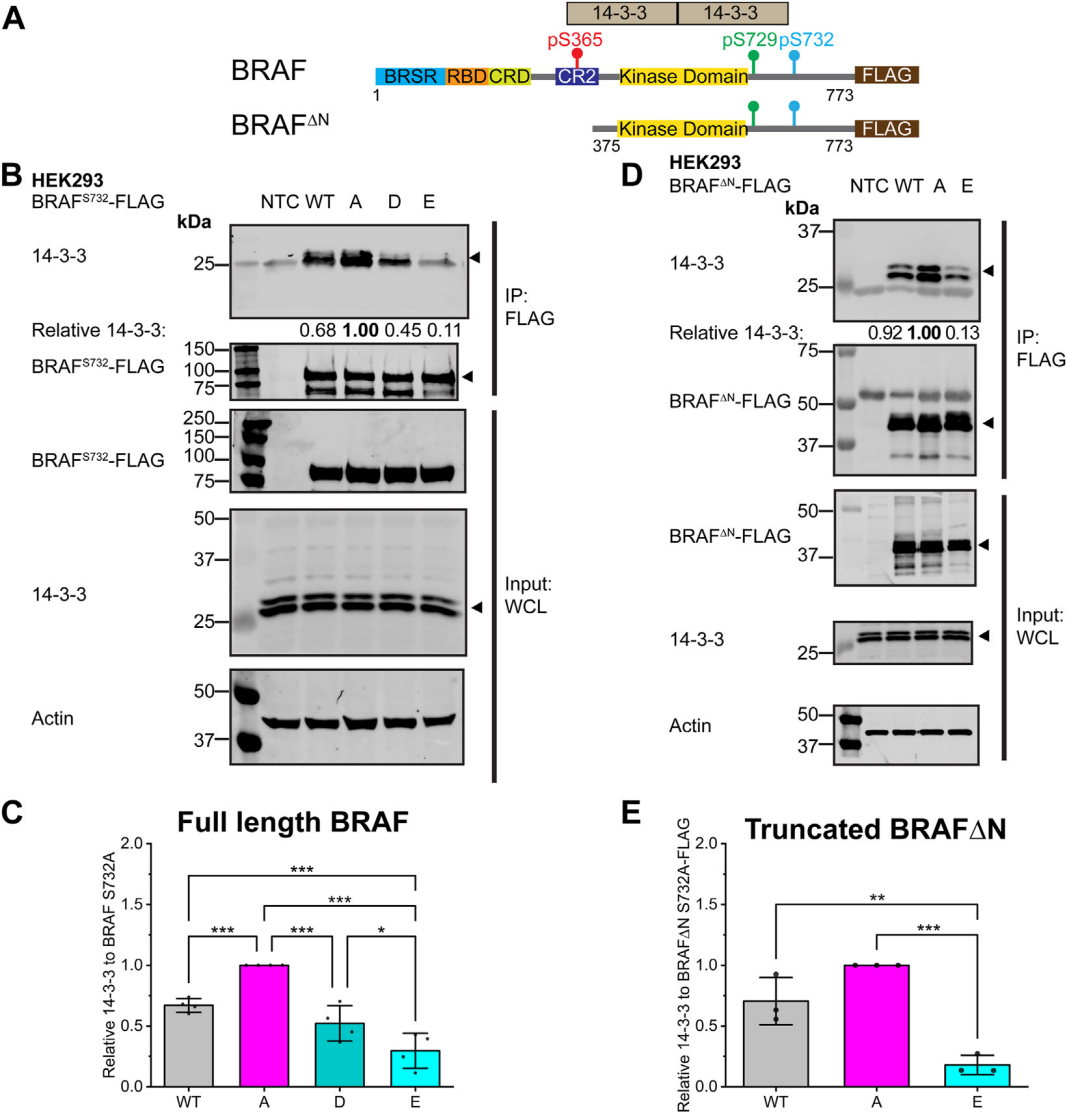
**BRAF**^**S732**^**phosphomimetic mutations decrease 14-3-3 scaffolding protein association with BRAF at the C-terminus region.***A*, cartoon schematic of the architecture of full-length BRAF and truncated BRAF^ΔN^ (aa 375–773). Truncated BRAF examines the C-terminus region’s local effects of BRAF^S732^ with the 14-3-3 scaffolding protein through the removal of S365. Full-length BRAF retains the interaction with 14-3-3 at the N- and C-terminal region, as indicated by the cartoon schematic of the 14-3-3 scaffolding protein dimer. *B*, representative immunoblot of the mutational analysis of full-length BRAF^S732^-FLAG of WT, phospho-deficient (S732A, labeled as *A*), and phosphomimetic (S732D/E, labeled as *D* or *E*, where indicated) in HEK293 cells. Exogenous BRAF-FLAG was expressed and immunoprecipitated (IP) and coimmunoprecipitated (co-IP) for the endogenous 14-3-3 interaction and normalized to the BRAF^S732A^ phospho-deficient mutant (relative 14-3-3 ratio). The phospho-deficient mutant has no available phospho-S732A site and is the model for the nonphosphorylated state. In the representative blots, the *triangle* indicates the specific band of interest in blots with multiple bands. The whole-cell lysate (WCL) is loaded as a control for the immunoprecipitation experiments. The nontransfected control (NTC) represents the background endogenous cell control. (n = 4). *C*, densitometry analysis of relative co-IP 14-3-3 to phospho-deficient BRAF^S732A^ in four biological replicates. *D*, representative immunoblot of IP truncated BRAF^ΔN^-FLAG with the co-IP 14-3-3 scaffolding protein. Relative to BRAF^ΔN S732A^, phosphomimetic BRAF^ΔN S732E^ significantly decreases its association with the 14-3-3 scaffolding protein (relative 14-3-3 is normalized to the A mutant). (n = 3). *E*, densitometry analysis of three biological replicates relative 14-3-3 co-IP to the truncated BRAF^ΔN S732A^. In all graphs, the bars represent the mean± SD. The *dots* on the bar graphs correspond to an individual data point per replicate. Statistical significance was determined *via* one-way ANOVA, followed by the Tukey’s HSD test. *p*-values are represented by: ∗*p* < 0.05, ∗∗*p* < 0.01, and ∗∗∗*p* < 0.001. The markers next to the immunoblots represent molecular weights in kDa. HSD, honest significant difference.

14-3-3 plays a dual role in the regulatory mechanism of RAF. Upstream RAS has been reported to facilitate the relief of autoinhibition by promoting events that dephosphorylate N-terminal region BRAF^S365^ to dissociate 14-3-3 (1). It is unclear how the C-terminal region dissociates from 14-3-3. BRAF^S732^ is located just after the C-terminal 14-3-3 recognition motif that contains phosphorylated BRAF^pS729^ (Fig. 1) (11). We hypothesized that phosphorylation of BRAF^S732^ could interfere with the C-terminal region 14-3-3 scaffolding protein association. Co-IP experiments using phosphomimetic mutants of BRAF^S732^ revealed that BRAF^S732D/E^ reduces 14-3-3 association relative to the phospho-deficient BRAF^S732A^ mutant (Fig. 2, *A*–*C*). BRAF^S732E^ was chosen as the representative phosphomimetic mutant since it can mimic the phosphate oxygens of a phosphorylated-serine due to the longer side chain length when compared to an aspartic acid (26). Both phosphomimetic D/E mutants were significantly different than the phospho-deficient A mutant (Fig. 2, *A*–*C*). Further experiments were carried out to investigate the local effects of 14-3-3 binding at the C terminus of BRAF, using a mutant lacking the N-terminal 14-3-3 (BRAF^pS365^) binding region (BRAF^ΔN^, aa 375–773, Fig. 2, *D* and *E*). Notably, BRAF^S732E^ displayed a highly significant decrease in C-terminal region 14-3-3 association (Fig. 2, *D* and *E*). Full-length BRAF^S732^ WT, A, and E mutants were expressed and immunoprecipitated from HELA and HCT116 cells (Fig. S1). HELA cells contain nonmutated endogenous MAPK components, while HCT116 contains constitutively active upstream KRAS^G12C^. In both cell lines, full-length phosphomimetic BRAF^S732E^ associates with the endogenous 14-3-3 scaffolding protein at a lower level than phospho-deficient BRAF^S732A^ (Fig. S1). Together, these data suggest that the dissociation of 14-3-3 with phosphomimetic BRAF^S732E^ is not cell line–specific.

**Figure 3:**
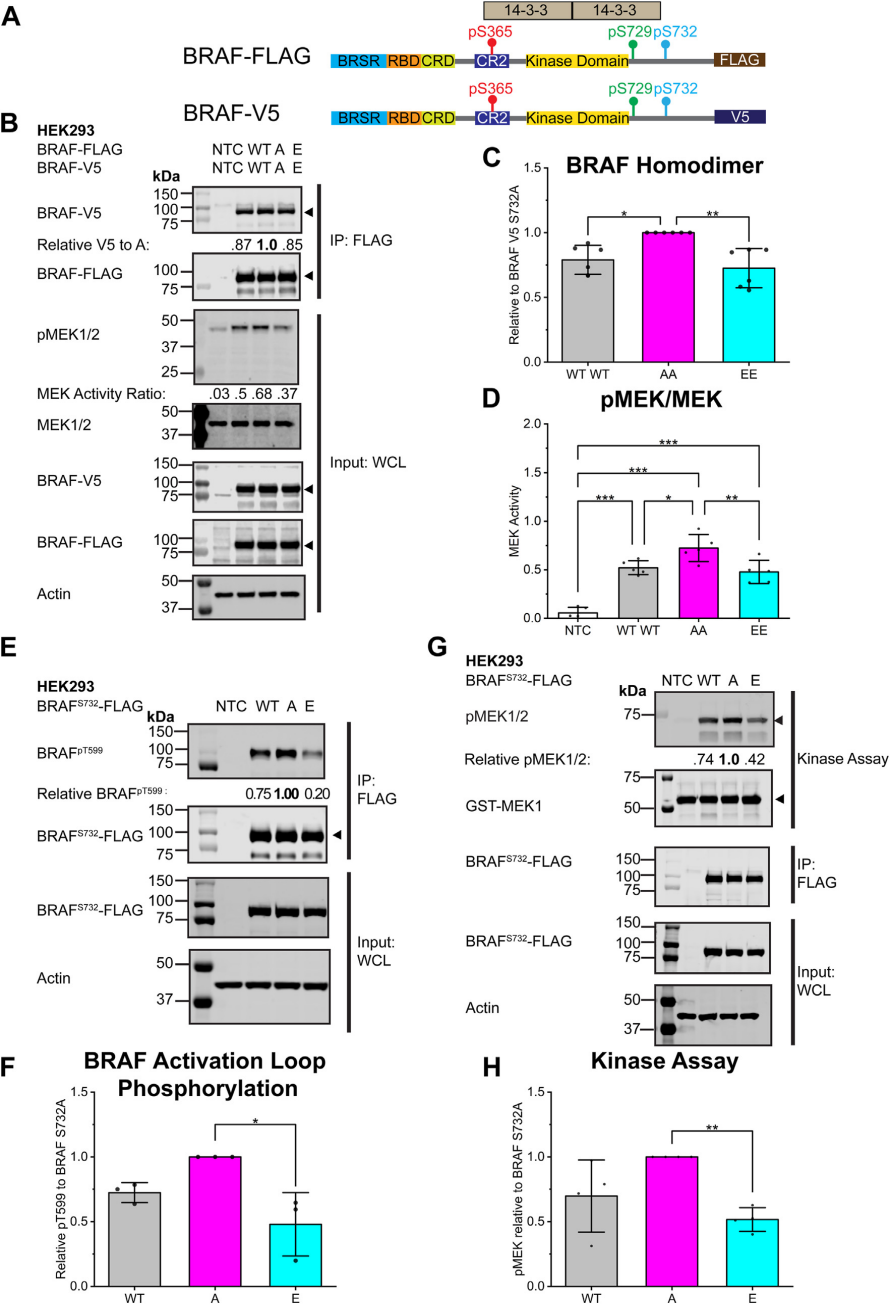
**Phosphomimetic BRAF**^**S732**^**decreases BRAF homodimerization, phosphorylation of activation loop, and phosphorylation of MEK.***A*, cartoon schematic of the full-length BRAF-FLAG and BRAF-V5 constructs used in the BRAF homodimer experiment. Both constructs contain BRAF S732 WT, S→A, or the S→E mutation. *B*, representative immunoblot of BRAF-FLAG and BRAF-V5 coexpressed in HEK293 cells to probe for the BRAF homodimer. Relative V5 ratio is normalized to the phospho-deficient A mutant. MEK1/2 is downstream of the RAF–MEK–ERK pathway. Active BRAF phosphorylates MEK1/2 (pMEK1/2), as seen in the whole-cell lysate (WCL). The MEK activity ratio is determined by phosphorylated MEK1/2/total MEK1/2 protein. (n = 6) NTC is the nontransfected cells to represent background cellular activity. *C*, densitometry analysis of the coimmunoprecipitated BRAF-V5 relative to BRAF^S723A^-V5. Phosphomimetic BRAF^S732E^ decreases BRAF-V5 association in comparison to the phospho-deficient A mutant in six biological replicates. Abbreviations of WT WT, AA, EE correspond to both protomers of the BRAF-FLAG and BRAF-V5 homodimer, respectively. *D*, densitometry analysis of the MEK activity ratio (phosphorylated/total protein) of six biological replicates. *E*, full-length BRAF-FLAG and mutant S732A/D/E mutants were expressed in HEK293 cells and FLAG immunoprecipitated BRAF-FLAG for its activation loop phosphorylation at pT599 (n = 3). *F*, densitometry analysis of three biological replicates for the activation loop phosphorylation normalized to the BRAF^S732A^. *G*, representative immunoblot-based kinase assay of four biological replicates (n = 4). Phosphorylated MEK is relative to the phospho-deficient BRAF^S732A^ mutant to highlight kinase activity. *H*, densitometry analysis of the relative phosphorylated MEK activity of BRAF WT, phospho-deficient BRAF^S732A^, and phosphomimetic BRAF^S732E^ mutants. Graph bars represent the mean± SD with individual data points per biological replicate. Statistical significance was determined *via* one-way ANOVA, followed by the Tukey’s HSD test. *p*-values are represented by: ∗*p* < 0.05, ∗∗*p* < 0.01, and ∗∗∗*p* < 0.001. The markers next to the immunoblots represent molecular weights in kDa. HSD, honest significant difference.

C-terminal region 14-3-3 has been shown to facilitate the dimerization of two RAF protomers (12). To model the BRAF homodimer, we coexpressed BRAF^S732 WT/A/E^-FLAG as protomer 1 and BRAF^S732 WT/A/E^-V5 as protomer 2; protomer 2 was coimmunoprecipitated using the FLAG epitope tag of protomer 1 (Fig. 3, *A* and *B*). Phosphomimicry of both protomers reduced BRAF homodimerization and MEK1/2 phosphorylation (Fig. 3, *B*–*D*). Dimerization is a key regulatory element of BRAF kinase activity. In immunoprecipitated BRAF-FLAG, phospho-deficient BRAF^S732A^-FLAG is observed to have a relatively higher activation loop phosphorylation (BRAF^pT599^) than BRAF^S732E^ (Fig. 3, *E* and *F*). The increased activation loop phosphorylation is also observed in HELA cells in the BRAF^S732A^ relative to BRAF^S732E^ (Fig. S1, *A* and *C*). In an *in vitro* Western blot–based kinase assay, *Escherichia coli* expressed GST-MEK1 was added to immunoprecipitated BRAF ^S732WT/A/E^-FLAG. Similar to the endogenous MEK1/2 phosphorylation in HEK293 cells, *in vitro* immunoprecipitated phosphomimetic BRAF^S732E^ phosphorylated MEK1 less relative to the phospho-deficient A mutant (Fig. 3, *H* and *I*). Phosphomimicry decreases local C-terminal region 14-3-3 scaffolding protein association and reduces BRAF homodimerization with observed reduced activation loop phosphorylation and downstream MEK1/2 phosphorylation.

### Dabrafenib differentially induces paradoxical activation of phospho-deficient BRAF^S732A^ and phosphomimetic BRAF^S732E^

**Figure 4:**
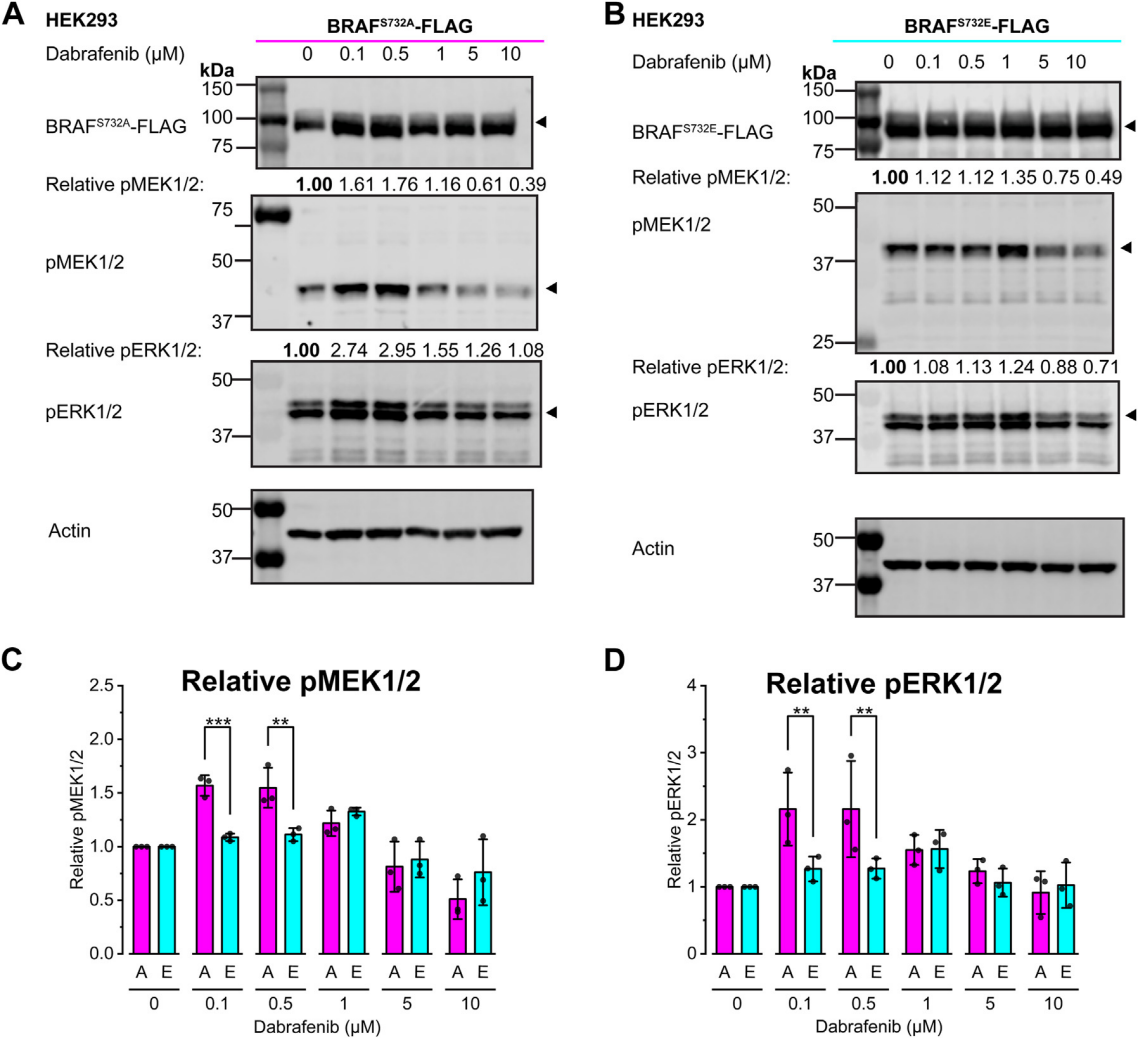
**Dabrafenib differentially induces paradoxical activation of phosphodeficient BRAF**^**S732A**^**and phosphomimetic BRAF**^**S732E**^**.***A* and *B*, representative immunoblot of three biological replicates expressing BRAF^S732A^-FLAG (*A*) and BRAF^S732E^-FLAG (*B*) in HEK293 cells with 1-h dabrafenib treatment (0–10 μM). Phosphorylated MEK1/2 (pMEK1/2) and phosphorylated ERK1/2 (pERK1/2) are included as RAF-MEK1/2-ERK1/2 phosphorylated activity readouts of the MAPK pathway in response to dabrafenib treatment (n = 3). The markers next to the immunoblots represent molecular weights in kDa. *C* and *D* densitometry analysis of three biological replicates for relative (*C*) pMEK1/2 and (*D*) pERK1/2 ratios. Relative ratios are normalized to the 0 μM (n = 3). The 0 μM accounts for the highest volume of DMSO. Phosphomimetic BRAF^S732E^ requires a higher dose of dabrafenib to induce paradoxical activation. Graph bars represent the mean± SD with individual data points per biological replicate. Statistical significance was determined via two-way ANOVA, followed by the post hoc Holm–Bonferroni test for multiple comparisons. *p*-values are represented by: ∗*p* < 0.05, ∗∗*p* < 0.01, and ∗∗∗*p* < 0.001.

Three FDA-approved inhibitors (dabrafenib, vemurafenib, and encorafenib) have been successful in targeting metastatic melanoma with BRAF^V600E/K^ mutants (14, 15); however, these inhibitors are less effective against non-BRAF^V600^ mutants (27). Paradoxical activation of RAF occurs with low dosage of dabrafenib and vemurafenib treatment in RAF-driven cancers and cell lines, presenting the paradox (3). Several theories have been proposed, such as enhanced dimerization of RAF protomers upon inhibitor binding, RAS-priming, and negative allostery, where an inhibitor-bound protomer can transactivate the inhibitor-free protomer (3). These models suggest that these FDA-approved inhibitors are not effective against dimeric RAF. Dabrafenib-induced paradoxical activation has been observed previously in overexpressed BRAF^WT^ in HEK293 cells in the 0.1 to 1 μM range (18). Short-term dabrafenib treatment of BRAF^S732A^ induces the classical paradoxical activation similar to BRAF^WT^ (Fig. 4, *A*, C and *D*). In contrast, BRAF^S732E^ required a higher concentration than BRAF^S732A^ to induce paradoxical activation, displaying enhanced MEK and ERK phosphorylation consistently at 1 μM dabrafenib treatment (Fig. 4, *C* and *D*). Phosphomimicry of BRAF^S732^ reduces homodimer formation, narrowing the range of paradoxical activation to 1 μM unlike phospho-deficient BRAF^S732A^, which has paradoxical activation observed at 0.1 to 1 μM. Phosphomimicry of BRAF^S732^ does not abolish paradoxical activation but rather, paradoxical activation requires a higher dosage to occur, likely due to the reduced homodimer formation in BRAF^S732E^ (Fig. 4).

### Phosphomimetic BRAF^S732E^ and CRAF^S624E^ decreases the BC heterodimer and decreases MAPK pathway activity by decreasing MEK phosphorylation in cells

**Figure 5:**
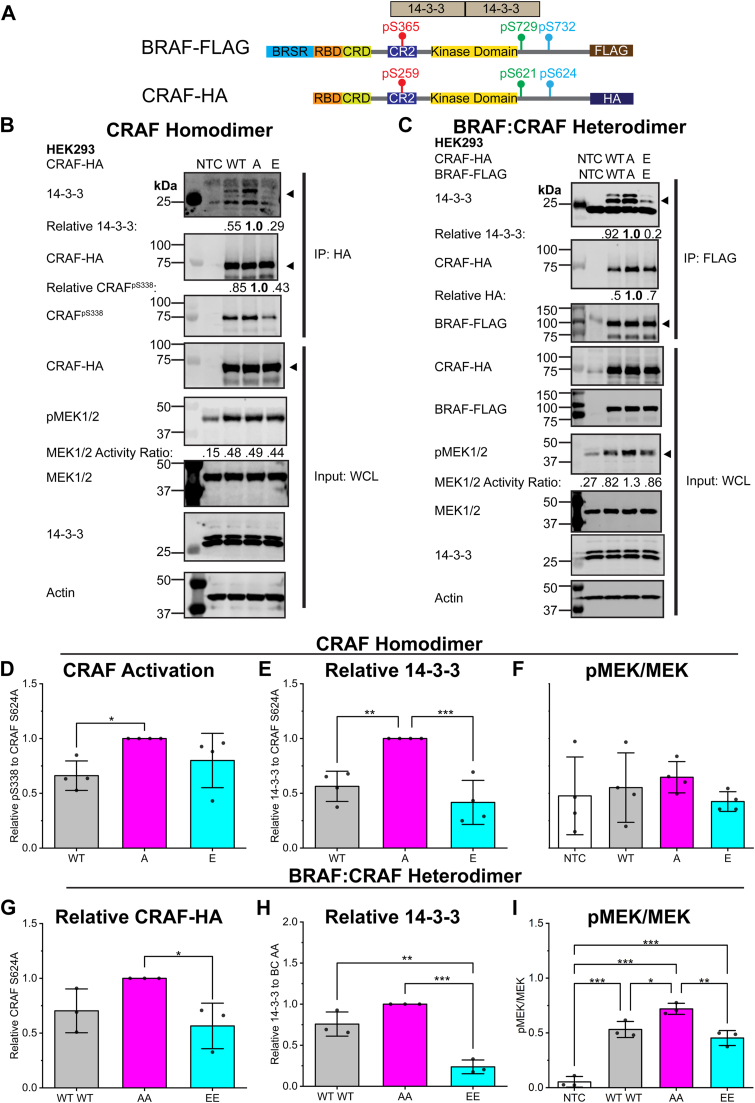
**Phosphomimetic mutants decrease the CRAF homodimer and BRAF:CRAF heterodimer and decrease MAPK signaling.***A*, BRAF-FLAG and CRAF-hemagglutinin (HA) constructs used for the CRAF homodimer and BRAF:CRAF heterodimer experiments. CRAF^S624^ is the homologous BRAF^S732^ site and is identically conserved. *B*, the CRAF-HA S624WT/A/E homodimer were expressed in HEK293 cells. CRAF-HA was immunoprecipitated (IP *via* HA) for the coimmunoprecipitation of endogenous 14-3-3 and endogenous MEK1/2 activity (phosphorylated/total protein). Representative blot of four biological replicates (n = 4). *C*, to model the BRAF:CRAF heterodimer, BRAF-FLAG, and CRAF-HA were coexpressed in HEK293 cells, where each protomer is solely WT/A/E. Representative immunoblot of CRAF associated with immunoprecipitated BRAF is shown from three biological replicates (n = 3). Relative 14-3-3 and CRAF-HA was normalized to the AA phospho-deficient BC heterodimer. *D*–*F*, densitometry analysis of the CRAF homodimer (*B*). Relative CRAF activation at pS338 (*D*) and 14-3-3 association (*E*) was normalized to the phospho-deficient A mutant. *F*, CRAF homodimer MEK activity (phosphorylated MEK1/2/total MEK1/2) was not significantly affected by mutational status. *G–I* densitometry analysis of the BRAF:CRAF heterodimer relative to the phospho-deficient AA model for CRAF association (*G*), relative 14-3-3 (*H*), and MEK1/2 activity (*I*). Statistical significance was determined *via* one-way ANOVA, followed by the Tukey’s HSD test. *p*-values are represented by: ∗*p* < 0.05, ∗∗*p* < 0.01, and ∗∗∗*p* < 0.001. HSD, honest significant difference.

The BRAF^S732^ phosphoregulatory site is conserved across the RAF family kinases in humans, with CRAF^S624^ being the conserved site (Fig. 1*A*). Phosphorylation of the CRAF^S624^ site has been previously annotated in the PhosphoSite database (21, 22, 23, 28). To investigate the effects of phosphorylation of S624 on CRAF, two mutants were created: CRAF^S624E^ to mimic phosphorylation and CRAF^S624A^ to abolish phosphorylation (Fig. 5, *A* and *B*). Consistent with the BRAF experiments, phospho-deficient CRAF^S624A^ displayed enhanced association with the 14-3-3 scaffolding protein (Fig. 5, *B* and *E*). To further probe the regulation of CRAF, we examined the phosphorylation status (CRAF^pS338^) of immunoprecipitated CRAF. CRAF is regulated by similar but distinct mechanisms compared with BRAF, with a CRAF SSYY motif (residues 338–341) instead of the BRAF SSDD motif (residues 446–449), the N-terminal acidic region (29, 30). Phosphorylation of CRAF^S338^ is critical for CRAF activation, RAS association, and dimerization (29, 30). We observed increased phosphorylation of CRAF^S338^ in immunoprecipitated CRAF^S624A^ compared to the WT but no significance for the phospho-deficient E mutant (Fig. 5, *B* and *D*). In CRAF, there is no significance in the phosphorylation of MEK1/2, unlike BRAF (Fig. 5, *B* and *F*), suggesting that CRAF homodimer is less sensitive to the phospho-status of Ser624.

Since the CRAF homodimer is not the major RAF dimer to regulate MAPK pathway, we investigated how this phosphorylation modification impacts BRAF:CRAF heterodimer, the most potent MAPK pathway output dimers (1). The BRAF:CRAF heterodimer was modeled by coexpressed BRAF-FLAG as protomer 1 with CRAF-hemagglutinin (HA) tag as protomer 2. The heterodimer was coimmunoprecipitated by the FLAG tag. Each protomer contained the phospho-deficient A or the phosphomimetic E mutant. Similar results were observed for the BRAF homodimer and the BC heterodimer. Phosphomimicry reduced 14-3-3 scaffolding protein association, BC heterodimerization, and reduced downstream MEK1/2 phosphorylation (Fig. 5, *C*, *G*–*I*). The CRAF homodimer alone does not affect the phosphorylation of MEK but combined with BRAF in the heterodimer, the decrease in MEK1/2 phosphorylation for the phosphomimetic mutants is highly significant (Fig. 5, *F* and *I*). This observation is consistent with the BC heterodimer having the highest MAPK pathway activity and that CRAF alone has low-basal activity.

### Mutant BRAF^S732F^ decreases BRAF homodimerization and has low-MEK activity

**Figure 6:**
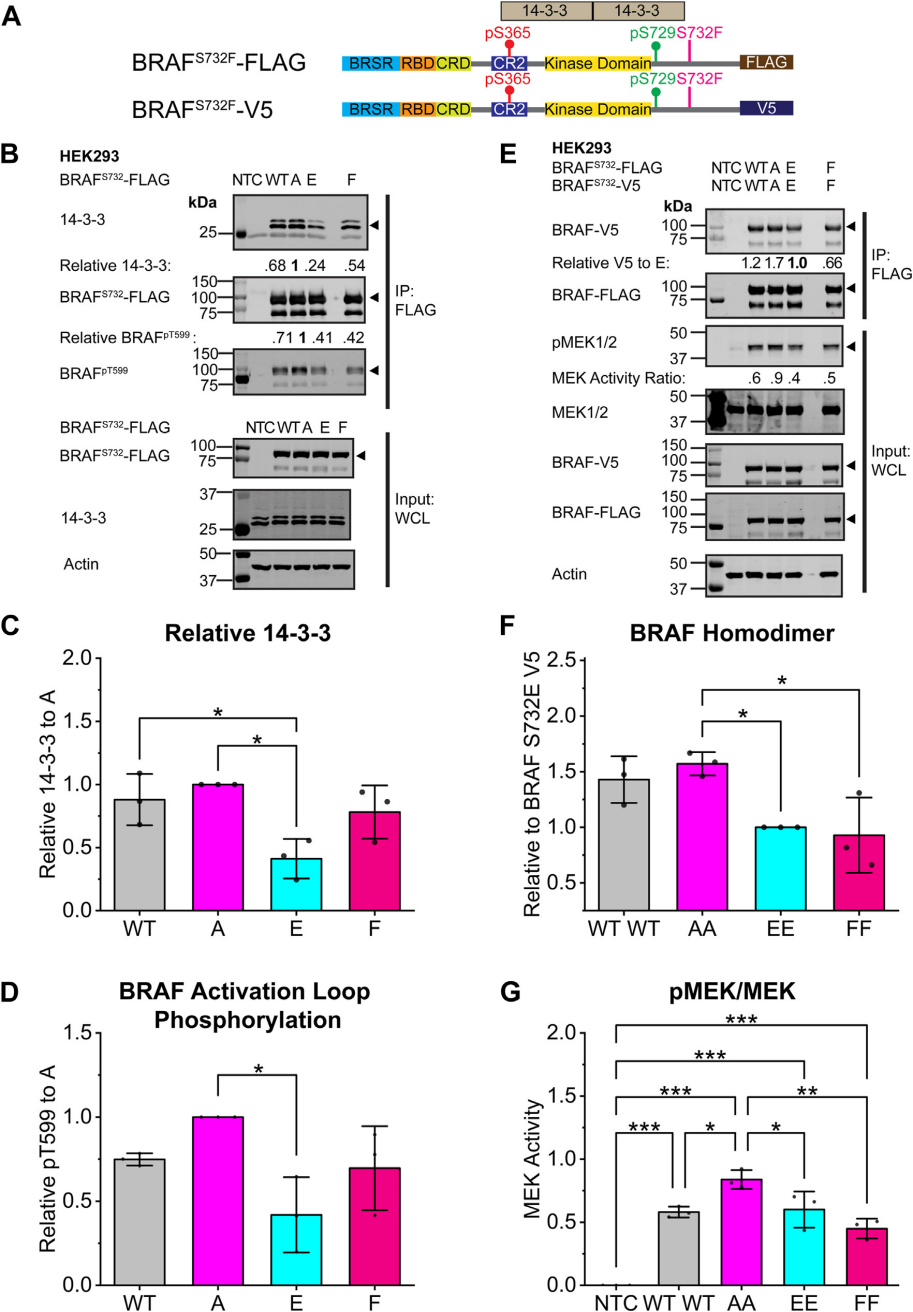
**Mutant BRAF**^**S732F**^**is in intermediate of the phospho-deficient and phosphomimetic model with decreased BRAF homodimerization and has low MEK activity.***A*, cartoon schematic of BRAF ^S732F^ with FLAG and V5 epitope tags. BRAF S732F is annotated in the COSMIC database with unknown biological effect. In *beige*, 14-3-3 associates with the N- and C-terminal region of BRAF at S365 and S729, respectively. *B*, analysis of the activation loop phosphorylation (BRAF pT599) and 14-3-3 association of BRAF^S732F^ in HEK293 cells. Representative immunoblot of immunoprecipitated BRAF-FLAG with coimmunoprecipitated 14-3-3 of three biological replicates for BRAF^S732F^ (n = 3). Relative 14-3-3 and BRAF^pT599^ ratio is normalized to BRAF^S732A^. *Black triangle* indicates the correct band in blots with multiple bands. *C*, densitometry analysis of 14-3-3 association with BRAF WT and BRAF S732→A/E/F mutants. *D*, densitometry analysis of three biological replicates for the activation loop phosphorylation (BRAF^pT599^) normalized to the BRAF^S732A^. *E*, phospho-deficient BRAF^S732A^ associates with the homodimer differently than BRAF^S732E/F^. The BRAF homodimer was expressed in HEK293 cells. BRAF-FLAG was immunoprecipitated for the BRAF-V5 protomer interaction to probe for the effect of the mutant on homodimer association (n = 3). Both protomers contain the WT, phospho-deficient (AA), the phosphomimetic (EE) mutations, or the COSMIC annotated (FF) mutations. Relative V5 ratios are relative to BRAF^S732E^ to emphasize the phospho-deficient model from being different from the EE and FF model. *Black triangle* indicates the correct band in blots with multiple bands. The MEK activity ratio is determined by the phosphorylation of MEK1/2/total MEK1/2 protein. *F*, densitometry analysis of the BRAF homodimer association relative to BRAF^S732E^-V5. Phospho-deficient A is significantly different from the phosphomimetic *E* and dysregulated *F* mutants. *G*, densitometry analysis of MEK activity across the BRAF homodimer experiments. BRAF^S732A^ is significantly different than the *E* and *F* mutants. All immunoblots contain their respective molecular weight markers where indicated. Statistical significance was determined *via* one-way ANOVA, followed by the Tukey’s HSD test. *p*-values are represented by: ∗*p* < 0.05, ∗∗*p* < 0.01, and ∗∗∗*p* < 0.001. HSD, honest significant difference.

While the focus of our analysis relied heavily on WT, phospho-deficient, and phosphomimetic BRAF and CRAF, many oncogenic cell lines contain mutations that dysregulate the RAF–MAPK pathway. BRAF^S732F^ is reported by the COSMIC database as a missense somatic mutation in carcinoma and malignant melanoma with an unknown effect (19, 20). Similar to BRAF^S732A^, phenylalanine lacks the phosphoacceptor site. However, due to its large size, the phenylalanine could also act as steric hinderance and could subscribe to either model. In HEK293, HCT116, and HELA cell lines, BRAF^S732F^ does not have a significant impact on the activation loop phosphorylation (Figs. 6 and S1). However, 14-3-3 association is enhanced in the HELA and HCT116 cell lines (Fig. S1). Both cell lines contain higher endogenous MAPK activity than the HEK293 cell line, which is the likely culprit for this observed difference. The BRAF homodimer was expressed in HEK293 cells with mutant F and compared to the phosphomimetic E. Both the phenylalanine mutant and the phosphomimetic E mutant are significantly different than the phospho-deficient A model (Fig. 6, *E* and *F*). BRAF^S732F^ decreases homodimerization in a similar manner to the phosphomimetic model, which further emphasized by the low MEK1/2 activity in HEK293 cells (Fig. 6, *E*–*G*). The 14-3-3 association is not significant in HEK293 cells, but mutant phenylalanine decreases BRAF homodimerization and MEK activity in a similar fashion to the phosphomimetic E model (Fig. 6, *E* and *F*). Based on decreased dimerization and MEK activity, we rationalized that mutant BRAF^S732F^ would respond to dabrafenib treatment similar to the phosphomimetic E model (Fig. S2). Both models under dabrafenib treatment were compared to BRAF^S732F^ and no significance was observed between the comparison of relative pMEK1/2 and pERK1/2 between the mutant F and E model (Fig. S2, *D* and *E*). BRAF^S732F^ behaves more similarly to the decreased MEK activity and decreased BRAF homodimerization observed in the phosphomimetic BRAF^S732E^ model. These observations suggest that dysregulated BRAF^S732F^ does not solely follow the phosphomimetic nor phospho-deficient model of regulation. BRAF^S732F^ is not significantly affected by 14-3-3 association nor activation loop phosphorylation compared to the A model; however, this mutant significantly decreases MEK phosphorylation and BRAF homodimerization. Mutant BRAF^S732F^ is dysregulated as it increases 14-3-3 association, while decreasing BRAF homodimerization and downstream MEK1/2 activity. The characterization of mutant phenylalanine further emphasizes that dysregulated RAF does not follow a clear model.

## Discussion

**Figure 7:**
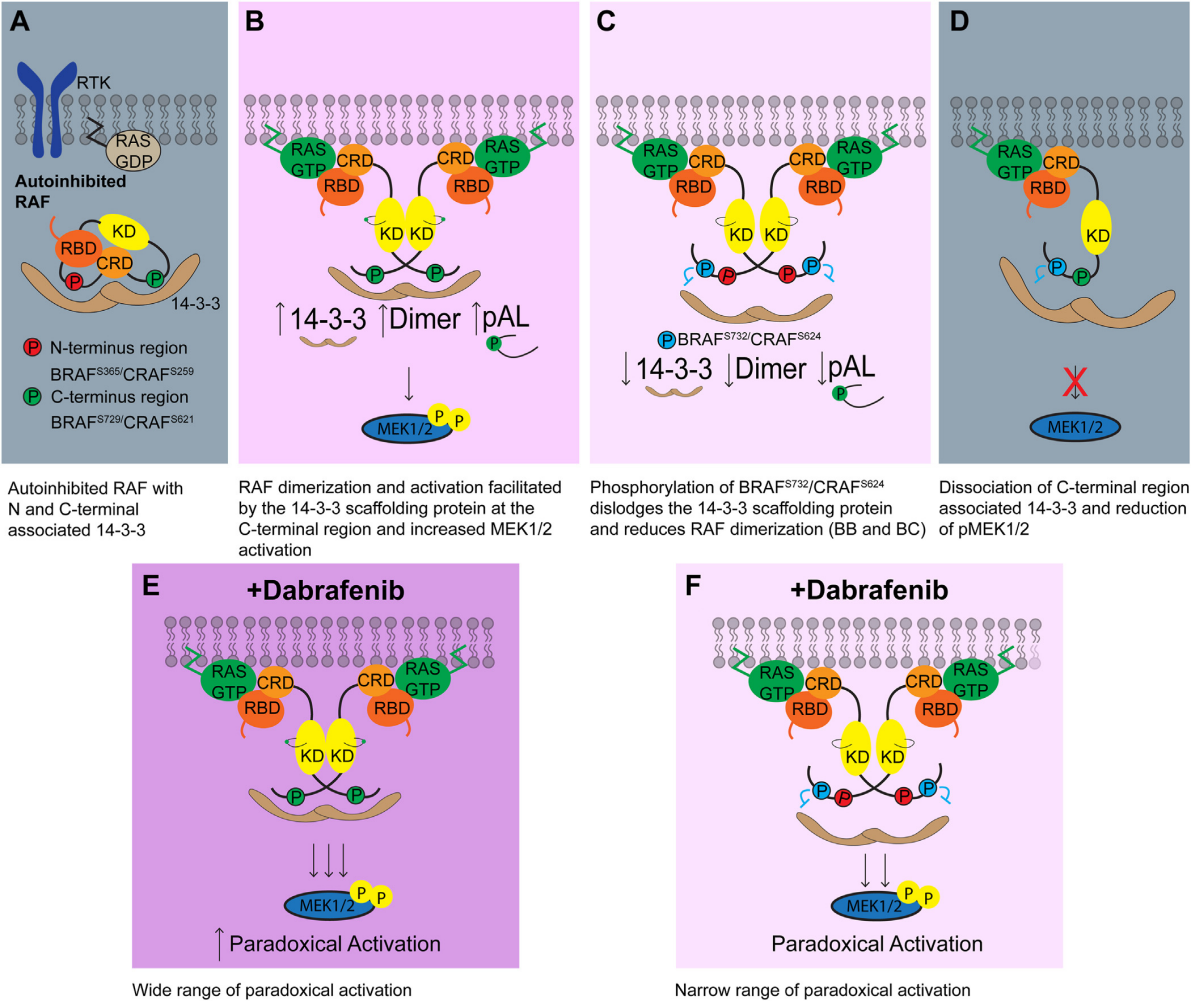
**Schematic of RAF regulation mechanism by novel phosphorylation site.***A*, N-terminal 14-3-3 scaffolding protein facilitates an autoinhibited conformation of BRAF. The C-terminus association with 14-3-3 facilitates the dimerization of two RAF protomers. *Green dots* symbolize activating phosphorylation sites and *red dots* are inactivating phosphorylation sites with respect to BRAF and CRAF. *B*, in normal cell signaling, BRAF^WT^/CRAF^WT^ likely exists in an equilibrium between the phosphomimetic E and phospho-deficient A models. Canonically, cytosolic, autoinhibited RAF (*A*) is recruited to the plasma membrane, where the RBD interacts with active RAS-GTP after RAF autoinhibitory relief. RAF enhances its association with the dimeric 14-3-3 scaffolding protein at the C-terminal region. Activated and dimerized RAF phosphorylates downstream MEK1/2. *C*, inversely, phosphorylation of BRAF^S732^/CRAF^S624^ leads to decreased 14-3-3 scaffolding protein association, activation loop phosphorylation, decreased BRAF homodimerization, and BC heterodimerization. *D*, phosphorylation of BRAF^S732^/CRAF^S624^ dislodges C-terminal 14-3-3. These negative regulatory consequences contribute to decreased MEK phosphorylation. Decreased MAPK activity returns the cells to basal level activity in which RAF is autoinhibited (*A*). *E* and *F*, while both models lead to increased MEK activation, the (*E*) phospho-deficient model has a wide range of paradoxical MEK1/2 activation than the (*F*) phosphomimetic model (*narrower range*).

Normal RAF kinase signaling is complex and tightly regulated with phosphorylation and dimerization being the key tenants of regulation to maintain proper MAPK signal transduction. BRAF^pS729^/CRAF^pS621^ is widely accepted to be constitutively phosphorylated and associated with 14-3-3 (6, 31). It has been unclear how the 14-3-3 scaffolding is regulated at the C terminus with constitutively phosphorylated BRAF^S729^. Our data suggest that the phosphorylation of BRAF^S732^ and CRAF^S624^ plays a negative regulatory role (Fig. 7). Outside of RAF, the 14-3-3 scaffolding proteins allosterically regulate the activity of many protein interactors in the cell (32). The identification of this negative regulatory mechanism for BRAF is not an uncommon strategy. Phosphorylation is known to indirectly modulate proximity interaction regions of the 14-3-3 scaffolding protein. This has been previously observed by the dual phosphorylation of the BH3-only protein at S128 to abrogate the regulatory 14-3-3 scaffolding protein interaction with S136; 14-3-3 dissociation enables proapoptotic BH3-only protein activity (33, 34). Phosphomimetic BRAF^S732E^ models a similar mechanism for disrupting the 14-3-3 scaffolding protein regulatory switch.

Mutational analysis identified a negative regulatory switch of BRAF^S732^ using phosphomimetic and phospho-deficient mutants to represent a phosphorylated and nonphosphorylated state of serine 732, which coordinates the 14-3-3 scaffolding protein association to modulate activity. Despite identical sequence conservation, ARAF was omitted due to its very low–kinase activity. BRAF^WT^ is represented by the two models, likely in equilibrium in cells: phospho-deficient BRAF^S732A^ and phosphomimetic BRAF^S732E^ (Fig. 7, *B* and *C*). During upstream stimulation *via* RAS-GTP, 14-3-3 association is enhanced, which increases homodimer and heterodimer formation, activation loop phosphorylation, and subsequently MEK activation (Fig. 7*B*). Subsequent phosphorylation of BRAF^pS732^ dislodges the association of the 14-3-3 scaffolding protein at the C terminus, which decreases dimerization, activation loop phosphorylation, and attenuation of MEK1/2 phosphorylation (Fig. 7, *C* and *D*). While CRAF is sensitive to the C-terminal region 14-3-3 association, no downstream consequences are observed. However, CRAF coupled with BRAF to form the BC heterodimer induces similar effects observed in the BRAF homodimer (Fig. 5, *C* and *I*). This data underscores the importance of the proper positioning of the two dimerized protomers of the RAF kinase domain with the 14-3-3 scaffolding protein as disruption has been previously demonstrated to disrupt RAF activity (31). Dimerization enforced by the 14-3-3 scaffolding protein acts as a flexibly bound molecular switch based on the observation of the kinase domain and 14-3-3 complex producing 500-fold difference in kinase activity in the presence of 14-3-3 proteins (35). C-terminus phosphorylation at BRAF^S732^/CRAF^S624^ finely tunes dimerization and MAPK activity by modulating 14-3-3 association to allosterically affect BRAF homodimer and BC heterodimer activity (Fig. 7). The finding of this novel site advances the understanding of the negative regulatory mechanism of RAF. Direct negative feedback phosphorylation by ERK on BRAF and CRAF has been previously reported on multiple sites to reduce RAF activity (36). Together, BRAF^S732^/CRAF^S624^- and ERK-mediated negative feedback phosphorylation sites jointly reduce the biological consequences of RAF signaling. The characterization of BRAF^S732^ and CRAF^S624^ introduces additional complexity to the negative regulatory mechanism of RAF.

In accordance with dimerization as a key driving force of paradoxical activation, impaired dimer formation of BRAF^S732E^ alters dabrafenib sensitivity (Fig. 7, *E* and *F*). How exactly RAF inhibitors affect the RAF:14-3-3 complex is not well defined and requires further understanding. This cell-based data provides an additional therapeutic target for reducing BRAF dimerization and activity. Two independent groups identify targeting the 14-3-3 interaction with BRAF as a site of therapeutic intervention as the scaffolding protein is important as a molecular switch (5, 31). Kondo *et al* proposed the α-I helix of BRAF as a potential site of therapeutic intervention based on the cryo-EM structures, making extensive interactions with 14-3-3 to interfere with RAF activity (31).

Our data supports C-terminal region 14-3-3 disruption as evidenced by the phosphomimetic BRAF^S732^–mediated reduction of downstream MEK1/2 phosphorylation. Disruption of protein–protein interaction by allostery have shown exceptional success in targeting RAF for MAPK downregulation as evidenced by peptides that impede RAF dimerization (18, 37). This identification of the novel phosphorylation site provides support for an alternative allosteric site for therapeutic intervention.

Due to the proximity of BRAF^S732^/CRAF^S624^ to the 14-3-3 recognition motif, phosphorylation of the site promotes the dissociation of the endogenous 14-3-3 scaffolding protein at the C terminus of RAF kinase. There are no commercially available phosphorylated BRAF^S732^ or CRAF^S624^ antibody to confirm the phosphorylation status of both sites. The effects upon the activation loop and many other available phosphorylation sites of RAF prevent the usage of a pan-phosphorylated serine antibody. We pursued mutational analysis of this novel phosphosite in BRAF and CRAF based on the identification submitted by other independent groups in addition to our mass spectrometry (MS) detection (28). Finally, the stoichiometry of the phosphorylated site remains unknown. Our model is built upon the population in which the stoichiometry of all exogenously expressed RAF to be phosphomimetic or phospho-deficient. Based on the analysis presented, phosphomimetic mutant dissociates 14-3-3 at the C terminus, which is in accordance with previous SPR data that the kinase domain of BRAF is flexibly bound to 14-3-3 (35). Our data supports that 14-3-3-mediated RAF-dimerization is a key regulatory mechanism in RAF signaling as disruption of 14-3-3 association decreased MEK phosphorylation. This phosphosite is a novel negative regulator of RAF activity through BRAF. Finally, the identified site supports therapeutic intervention at the C-terminus association with 14-3-3 as negative regulation decreases MAPK signaling.

## Experimental procedures

### Antibodies

Anti-Flag (F1804) was purchased from Sigma. Anti-pBRAF^T599^ (PA5-37497) was purchased from Invitrogen. Anti-HA (3724), anti-pMEK^S217/S222^ (9154), anti-MEK (9154), anti-pERK^T202/Y204^ (4370), ERK1/2 (4696), anti-β-Actin (3700), anti-pan 14-3-3 monoclonal (8312), anti-pan 14-3-3 polyclonal (95,422), anti-pCRAFS338 (9427) was purchased from Cell Signaling. Anti-GST was purchased from Santa-Cruz (SC-138). Anti-rabbit (926–32211) and anti-mouse (926–68070) secondary antibodies were purchased from LICOR.

### Molecular biology techniques and plasmid construction

All expression vectors are in the transient expression vector, pcDNA4/TO (Invitrogen V102020). BRAF-FLAG was cloned into the pcDNA4/TO as previously described (38). The primers used for the cloning are as follows in standard notation (5’->3′) and listed as forward and reverse primers, respectively: BRAF S732A (ATCAGAACCCGCCTTGAATCGGG, GCACTGCGGTGAATTTTTG), BRAF S732D (ATCAGAACCCGACTTGAATCGGGC, GCACTGCGGTGAATTTTTG), BRAF S732E (ATCAGAACCCGAATTGAATCGGGCTG, GCACTGCGGTGAATTTTTG), BRAF S732F (TCAGAACCCTtCTTGAATCGG, TGCACTGCGGTGAATTTTTG), BRAF ΔN (GAACCTGTCAATATTGATG, CATGGTAAGCTTAAGTTTAAAC), CRAF S624A (TTCCGAGCCAGCCTTGCATCGGG, GCGCTCCGGTTGATCTTCGG), CRAF S624E (TTCCGAGCCAGAATTGCATCGGGC, GCGCTCCGGTTGATCTTC). The BRAF S732F mutation was selected as a C2195T mutation to model the annotation in the COSMIC database. Constructs were created using standard cloning techniques *via* PCR, following the manufacturer’s instructions, Q5 site–directed mutagenesis (NEB E0554). Mini preps were carried using the Qiagen (27,106) mini prep kit according to the manufacturer’s protocols. Midi preps for transfection were carried out using the ZymoPURE (D4201) midi prep purification kit according to the manufacturer’s protocols. DNA elutions were carried in water.

### Cell culture, transfection, and dabrafenib treatment/co-IP

HEK293 cells (gift from Dr Catherine Moore, University of the Sciences) were maintained at 37 °C, 5% CO_2_ until 70 to 90% confluency and passaged until passage 16 and discarded. Cells were maintained in Dulbecco’s Modified Eagle Medium supplemented with 1% L-Glutamate, 1% penicillin and streptomycin, and 10% fetal bovine serum.

For co-IP experiments, cells were seeded with 5 million cells per 100-mm dish and transfected at 60% confluency with a 1:3 ratio of DNA:transfectant (Lipofectamine 2000, Invitrogen 11668-019, or PEI-MAX). Single transfections were carried with 10 μg in a 100-mm dish. The BRAF homodimer was carried using a 1:1 ratio of BRAF-FLAG: BRAF-V5. The BC heterodimer was carried using a 1:2 ratio of BRAF:CRAF due to the low expression of CRAF Cells 40 to 60% confluency before transfection. Transfections were cultured for 48 h at 37 °C, 5% CO_2_ before harvest using cold PBS. Cells were lysed using a modified radioimmunoprecipitation assay buffer. Cells were lysed using a modified radioimmunoprecipitation assay buffer (50 mM Hepes pH 7.4, 150 mM NaCl, 0.1% NP40, 1 mM EDTA, 5% glycerol, 1 mM PMSF, 20 mM β-glycerophosphate, 2.5 mM sodium pyrophosphate, protease inhibitor (Roche 11836170001)) at 4 °C, 2 h rotation. Immunoprecipitation was carried according to manufacturer’s protocols for the immunoprecipitation of FLAG-tagged proteins (Sigma M8823) and quenched in 4× Laemmli loading dye for 5 min at 95 °C)

Single transfections were carried with 2 μg/well in a 6-well plate (1 million per well) for the dabrafenib experiments. Following 48 h of protein expression, the cells were washed with PBS and replenished with 2 ml of fresh media. Dabrafenib (Selleckchem S2807) was diluted to a final concentration of 0.1 to 10 μM, using a 100 μl treatment volume. The negative control 0 μM contained the same volume of dimethyl sulfoxide as the 10 μM treatment. The 0 control contains the highest dilution volume of dabrafenib to account for effects of DMSO, all under 1% DMSO. Cells were incubated at 37 °C, 1 h prior to cell harvest. Cells were washed with PBS twice and lysed immediately with 4% SDS and subsequently applied to a homogenizer column.

### Immunoblot

Whole-cell lysate protein quantification was carried using a Pearce bicinchoninic acid assay and a Clariostar plate reader (BMG). Samples were diluted in 4× Laemmli loading dye and boiled for 5 min. Samples were visualized using 10% SDS-PAGE electrophoresis or 4 to 15% Mini-PROTEAN TGX Precast Protein Gels (Bio-Rad 4561084). Membranes were incubated in primary antibodies, overnight in 4 °C and secondary antibody 2 h, at room temperature. Nitrocellulose membranes were visualized using a LICOR scanner, and images were cropped to highlight the molecular weight marker, which is not visible on each scanning channel. For these channels, the molecular weight is placed next to the expected band size. If there are multiple bands beyond the expected molecular weight, a black arrow was placed to the right of the blot to indicate the correct band(s). Each reported experiment was performed three or more times, as indicated.

### Densitometry analysis

Densitometry quantification analysis was completed on ImageJ (https://imagej.nih.gov/ij/download.html) (NIH) through manual selection of the area under the curve (AUC) of the density of pixels. The AUC was normalized relative to the A mutant or the 0 μM treatment. For example, relative 14-3-3 to A mutant: AUC of sample/AUC of A mutant. This was chosen to highlight the dichotomy between the A and the E mutant. The following readouts use the same relative to A mutant method: 14-3-3, BRAF pT599, CRAF pS338, dimer readouts (BRAF-V5 relative to BRAF^S732A^-V5 of the BRAF homodimer and CRAF-HA relative to CRAF^S624A^-HA of the BRAF-CRAF heterodimer), and the *in vitro* kinase assay. For the kinase assay, relative phosphorylated MEK was relative to the phospho-deficient A mutant to reflect kinase activity between mutants. For the BRAF homodimer, CRAF homodimer, and the BRAF:CRAF heterodimer, endogenous phosphorylated MEK1/2 was normalized to the total MEK1/2 protein to reflect the population of MEK that is phosphorylated in the cells. Relative pMEK: phosphorylated MEK1/2/total MEK1/2. Dabrafenib treatments were normalized to the 0 μM treatment of the phosphorylated MEK. Previous reports (18, 39) use this densitometry normalization method as it reflects the change of MEK phosphorylation in response to dabrafenib treatment to highlight paradoxical activation of BRAF.

### Statistical analysis of immunoblots

All experiments were at least three independent biological replicates. Experimental differences were tested for statistical significance using one-way ANOVA followed by the post hoc Tukey’s honest significant difference test for co-IP experiments. Two-way ANOVA was used for the dabrafenib experiments with mutant BRAF and dabrafenib as the factors followed by the post hoc Holm–Bonferroni test for multiple comparisons (40). Statistical analysis was performed and plotted using the OriginLab (https://www.originlab.com/) (2023b v 10.05) software. Data points are within the bar graphs; bars are represented as mean ±SD and statistical significance as: ∗*p* < 0.05, ∗∗*p* < 0.01, and ∗∗∗*p* < 0.001. A *p*-value of <0.05 was considered statistically significant. All ANOVA values are reported in Supporting information Table S3.

### Kinase dead MEK1^K97M^ purification

Purification of recombinant kinase dead MEK1 (UniProt Q02750) was adapted from a previous report (41). In brief, kinase dead MEK1^K97M^ was expressed in *E. coli* BL21 codon+ with 6×HIS and GST tags in culture flasks until 0.6 to 0.8 *A* and induced with 0.5 mM IPTG for protein expression at 16 °C, 200 RPM rotation overnight. The cells were harvested, washed with PBS, and lysed in lysis buffer (20 mm Hepes pH 7.4, 150 mm NaCl, 10 mmβ-mercaptoethanol, 5 mM imidazole, 5% glycerol, 1 mg/ml lysozyme, and a protease inhibitor [Roche 11836170001]) and mechanical lysis *via* French press. Lysed supernatant of 6×HIS-kinase dead MEK1 was purified using buffer equilibrated nickel resin (1 ml, 2-h incubation). The resin was washed in sequential alternating low- and high-salt buffers to remove nonspecific binding (150 mM NaCl [low salt] or 500 mM NaCl [high salt], 20 mM Hepes pH 7.4, and 5% glycerol. MEK1 was eluted using 200 mM imidazole (150 mM NaCl, 20 mM Hepes pH 7.4).

### Immunoblot-based kinase assay

The kinase reaction was carried out using an adapted protocol as previously described (41). Immunoprecipitated BRAF-FLAG (using magnetic FLAG beads) from HEK293 cells was carried in a reaction buffer (50 mM Hepes pH 7.4, 10 mM MgCl_2_, 1 mM DTT, 25 mM β-glycerol phosphate, 1 μM recombinant kinase dead GST-MEK1^K97M^, and 1 mM ATP). The reaction incubated at 30 °C for 3.5 min was immediately quenched in 4× loading dye. The kinase reaction was visualized using the phosphorylated-MEK^S217/S22^antibody (Cell signaling 9154) and anti-GST (Santa Cruz SC-138) for the recombinant 6×HIS-GST-MEK1^K97M^.

### Mass spectrometry

Excised bands of basal, cytosolic BRAF, and membrane recruited, activated BRAF were excised and sent to the Proteomics and Mass Spectrometry Facility, University of Georgia for post translational modification identification (Thermo Fisher Scientific LTQ Orbitrap Elite MS with a Proxeon Easy NanoLC system). Briefly, gel bands were followed by an in-gel trypsin digestion. Extracted peptides were dried *via* SpeedVac and loaded into a reverse phase C18 column. Samples were directly eluted into the mass spectrometer using a two-buffer gradient elution (buffers A and B) at a flow rate of 450 nL/min. Buffer A was 0.1% formic acid and buffer B was 99.9% acetonitrile in 0.1% formic acid. Elution was carried using 0% buffer B (2 minutes), 30% buffer B (50 minutes), 50% buffer B (10 minutes), and finally 95% buffer B (10 minutes). The data-dependent acquisition method was used to acquire MS and tandem mass spectrometry (MS/MS) data (120,000 and 15,000 resolution, respectively) through the Xcalibur software (Thermo Fisher Scientific, https://www.thermofisher.com/us/en/home/industrial/mass-spectrometry/liquid-chromatography-mass-spectrometry-lc-ms/lc-ms-software/lc-ms-data-acquisition-software/xcalibur-data-acquisition-interpretation-software.html) and the UniProt database. Peptides were identified *via* Proteome Discoverer (Thermo Fisher Scientific). The data is available in Supplemental information 1-2. Previously reported phosphorylation site identification of BRAF S732 is available through the PhosphoSite database curation sets: 12,432, 9879, and 9883 (18).

## Data availability

The data that support the findings of this study are available on request from the corresponding author, Zhihong Wang, upon reasonable request.

## Supporting information

This article contains supporting information.

## Conflict of interest

The authors declare that they have no conflicts of interest with the contents of this article.

## References

[1] Lavoie H., Therrien M. (2015). Regulation of RAF protein kinases in ERK signalling. Nat. Rev. Mol. Cell Biol..

[2] Matallanas D., Birtwistle M., Romano D., Zebisch A., Rauch J., von Kriegsheim A. (2011). Raf family kinases: old dogs have learned new tricks. Genes Cancer.

[3] Karoulia Z., Gavathiotis E., Poulikakos P.I. (2017). New perspectives for targeting RAF kinase in human cancer. Nat. Rev. Cancer.

[4] Terrell E.M., Durrant D.E., Ritt D.A., Sealover N.E., Sheffels E., Spencer-Smith R. (2019). Distinct binding preferences between Ras and Raf family members and the impact on oncogenic Ras signaling. Mol. Cell.

[5] Park E., Rawson S., Li K., Kim B.W., Ficarro S.B., Pino G.G. (2019). Architecture of autoinhibited and active BRAF–MEK1–14-3-3 complexes. Nature.

[6] Molzan M., Schumacher B., Ottmann C., Baljuls A., Polzien L., Weyand M. (2010). Impaired binding of 14-3-3 to C-RAF in noonan syndrome suggests new approaches in diseases with increased Ras signaling. Mol. Cell Biol..

[7] Molzan M., Ottmann C. (2012). Synergistic binding of the phosphorylated S233- and S259-binding sites of C-RAF to One 14-3-3ζ dimer. J. Mol. Biol..

[8] Röring M., Herr R., Fiala G.J., Heilmann K., Braun S., Eisenhardt A.E. (2012). Distinct requirement for an intact dimer interface in wild-type, V600E and kinase-dead B-Raf signalling. EMBO J..

[9] Freeman A.K., Ritt D.A., Morrison D.K. (2013). The importance of Raf dimerization in cell signaling. Small GTPases.

[10] Shen C.H., Yuan P., Perez-Lorenzo R., Zhang Y., Lee S.X., Ou Y. (2013). Phosphorylation of BRAF by AMPK impairs BRAF-KSR1 association and cell proliferation. Mol. Cell.

[11] Noble C., Mercer K., Hussain J., Carragher L., Giblett S., Hayward R. (2008). CRAF autophosphorylation of serine 621 is required to prevent its proteasome-mediated degradation. Mol. Cell.

[12] Liau N.P.D., Venkatanarayan A., Quinn J.G., Phung W., Malek S., Hymowitz S.G. (2020). Dimerization induced by C-terminal 14-3-3 binding is sufficient for BRAF kinase activation. Biochemistry.

[13] Zhang M., Jang H., Li Z., Sacks D.B., Nussinov R. (2021). B-Raf autoinhibition in the presence and absence of 14-3-3. Structure.

[14] Bollag G., Hirth P., Tsai J., Zhang J., Ibrahim P.N., Cho H. (2010). Clinical efficacy of a RAF inhibitor needs broad target blockade in BRAF-mutant melanoma. Nature.

[15] Tsai J., Lee J.T., Wang W., Zhang J., Cho H., Mamo S. (2008). Discovery of a selective inhibitor of oncogenic B-Raf kinase with potent antimelanoma activity. Proc. Natl. Acad. Sci. U. S. A..

[16] Holderfield M., Deuker M.M., McCormick F., McMahon M. (2014). Targeting RAF kinases for cancer therapy: BRAF-mutated melanoma and beyond. Nat. Rev. Cancer.

[17] Samatar A.A., Poulikakos P.I. (2014). Targeting RAS-ERK signalling in cancer: promises and challenges. Nat. Rev. Drug Discov..

[18] Gunderwala A.Y., Nimbvikar A.A., Cope N.J., Li Z., Wang Z. (2019). Development of allosteric BRAF peptide inhibitors targeting the dimer interface of BRAF. ACS Chem. Biol..

[19] Bonilla X., Parmentier L., King B., Bezrukov F., Kaya G., Zoete V. (2016). Genomic analysis identifies new drivers and progression pathways in skin basal cell carcinoma. Nat. Genet..

[20] Krauthammer M., Kong Y., Ha B.H., Evans P., Bacchiocchi A., McCusker J.P. (2012). Exome sequencing identifies recurrent somatic RAC1 mutations in melanoma. Nat. Genet..

[21] Olsen, J. V, Vermeulen, M., Santamaria, A., Kumar, C., Miller, M. L., Jensen, L. J., et al. Quantitative Phosphoproteomics Reveals Widespread Full Phosphorylation Site Occupancy during Mitosis 10.1126/scisignal.2000475 20068231

[22] Christensen G.L., Kelstrup C.D., Lyngsø C., Sarwar U., Bøgebo R., Sheikh S.P. (2010). Quantitative phosphoproteomics dissection of seven-transmembrane receptor signaling using full and biased agonists. Mol. Cell Proteomics.

[23] Bian Y., Song C., Cheng K., Dong M., Wang F., Huang J. (2014). An enzyme assisted RP-RPLC approach for in-depth analysis of human liver phosphoproteome. J. Proteomics.

[24] Baljuls A., Schmitz W., Mueller T., Zahedi R.P., Sickmann A., Hekman M. (2008). Positive regulation of A-RAF by phosphorylation of isoform-specific hinge segment and identification of novel phosphorylation sites. J. Biol. Chem..

[25] Martinez Fiesco J.A., Durrant D.E., Morrison D.K., Zhang P. (2022). Structural insights into the BRAF monomer-to-dimer transition mediated by RAS binding. Nat. Commun..

[26] Pérez-Mejías G., Velázquez-Cruz A., Guerra-Castellano A., Baños-Jaime B., Díaz-Quintana A., González-Arzola K. (2020). Exploring protein phosphorylation by combining computational approaches and biochemical methods. Comput. Struct. Biotechnol. J..

[27] Holderfield M., Nagel T.E., Stuart D.D. (2014). Mechanism and consequences of RAF kinase activation by small-molecule inhibitors. Br. J. Cancer.

[28] Hornbeck P.V., Zhang B., Murray B., Kornhauser J.M., Latham V., Skrzypek E. (2015). PhosphoSitePlus, 2014: mutations, PTMs and recalibrations. Nucleic Acids Res..

[29] Takahashi M., Li Y., Dillon T.J., Kariya Y., Stork P.J.S. (2017). Phosphorylation of the C-Raf N region promotes Raf dimerization. Mol. Cell Biol..

[30] Hu J., Stites E.C., Yu H., Germino E.A., Meharena H.S., Stork P.J.S. (2013). Allosteric activation of functionally asymmetric RAF kinase dimers. Cell.

[31] Kondo Y., Ognjenović J., Banerjee S., Karandur D., Merk A., Kulhanek K. (2019). Cryo-EM structure of a dimeric B-Raf:14-3-3 complex reveals asymmetry in the active sites of B-Raf kinases. Science (1979).

[32] Morrison D.K. (2009). The 14-3-3 proteins: integrators of diverse signaling cues that impact cell fate and cancer development. Trends Cell Biol..

[33] Donovan N., Becker E.B.E., Konishi Y., Bonni A. (2002). JNK phosphorylation and activation of bad couples the stress-activated signaling pathway to the cell death machinery. J. Biol. Chem..

[34] Wang X.T., Pei D.S., Xu J., Guan Q.H., Sun Y.F., Liu X.M. (2007). Opposing effects of Bad phosphorylation at two distinct sites by Akt1 and JNK1/2 on ischemic brain injury. Cell Signal..

[35] Liau N.P.D., Wendorff T.J., Quinn J.G., Steffek M., Phung W., Liu P. (2020). Negative regulation of RAF kinase activity by ATP is overcome by 14-3-3-induced dimerization. Nat. Struct. Mol. Biol..

[36] Dougherty M.K., Müller J., Ritt D.A., Zhou M., Zhou X.Z., Copeland T.D. (2005). Regulation of Raf-1 by direct feedback phosphorylation. Mol. Cell.

[37] Beneker C.M., Rovoli M., Kontopidis G., Röring M., Galda S., Braun S. (2019). Design and synthesis of type-IV inhibitors of BRAF kinase that block dimerization and overcome paradoxical MEK/ERK activation. J. Med. Chem..

[38] Cope N., Candelora C., Wong K., Kumar S., Nan H., Grasso M. (2018). Mechanism of BRAF activation through biochemical characterization of the recombinant full-length protein. ChemBioChem.

[39] Cope N.J., Novak B., Liu Z., Cavallo M., Gunderwala A.Y., Matthew Connolly Z.W. (2020). Analyses of the oncogenic BRAF D594G variant reveal a kinase-independent function of BRAF in activating MAPK signaling. J. Biol. Chem..

[40] Fauser J., Huyot V., Matsche J., Szynal B.N., Alexeev Y., Kota P. (2022). Dissecting protein tyrosine phosphatase signaling by engineered chemogenetic control of its activity. J. Cell Biol..

[41] Luo C., Xie P., Marmorstein R. (2008). Identification of BRAF inhibitors through in silico screening. J. Med. Chem..

