# Supplementary material for: A novel phosphorylation site involved in dissociating RAF kinase from the scaffolding protein 14-3-3 and disrupting RAF dimerization
Source: J Biol Chem. 2023 Aug 23;299(10):105188. doi: 10.1016/j.jbc.2023.105188 (PMC10520314; doi:10.1016/j.jbc.2023.105188)
Supplement: Supporting information [file mmc1.docx]

**Supporting Information**

**Supporting information 1. Table S1.** List of all identified phospho-sites of BRAF and location across two samples sets of cytosolic and membrane recruited BRAF, captured by immunoprecipitation and identified via LC-MS/MS. BRAF^S732^, highlighted in bold and red. This table can be found as a separate xlsx file (Table SI1).

**Supporting information 2.** List of all identified phospho-sites of BRAF and location across two samples sets. Cytosolic BRAF-FLAG was membrane recruited and captured using immunoprecipitation. Post translational modifications of cytosolic and membrane localized BRAF-FLAG were identified using affinity-captured LC-MS/MS. BRAF^S732^, highlighted in bold and red, was identified along with other reported known and characterized phosphorylation sites in two biological replicates. This table can also be found as a separate xlsx file (Table SI2).

| **Sample** | **Phosphopeptide** | **Location on BRAF** |
| --- | --- | --- |
| Sample G, cytosolic BRAF | RDsSDDWEIPDGQITVGQR | S446 |
|  | SSsSSEDRNR | S430 |
|  | IGSGsFGTVYK | S467 |
|  | SAsEPSLNR | S729 |
|  | SNPKsPQKPIVR | S151 |
|  | IHRSAsEPSLNR | S729 |
|  | GYLsPDLSKVR | S675 |
|  | SRWsGSHQFEQLSGSILWMAPEVIR | S605 |
|  | DRSSsAPNVHINTIEPVNIDDLIR | S365 |
|  | sPQKPIVR | S151 |
|  | SRWsGSHQFEQLSGSILWMAPEVIR | S605 |
|  | DRSSsAPNVHINTIEPVNIDDLIR | S365 |
|  | sPQKPIVR | S151 |
|  | ALQKsPGPQR | S419 |
|  | SSsAPNVHINTIEPVNIDDLIR | S365 |
|  | DQGFRGDGGsTTGLSAtPPASLPGSLTNVK | S394, T401 |
|  | sSSAPNVHINTIEPVNIDDLIRDQGFR | S363 |
|  | KSsSSSEDR | S429 |
|  | FFEHHPIPQEEASLAETALTSGSSPsAPASDSIGPQILTSPSPSK | S319 |
|  | sNPKsPQKPIVR | S151 |
|  | sPGPQR | S419 |
| Sample G, membrane recruited BRAF | IGSGsFGTVYK | S467 |
|  | RDsSDDWEIPDGQITVGQR | S446 |
|  | DQGFRGDGGSTTGLSAtPPASLPGSLTNVK | T401 |
|  | ALQKsPGPQR | S419 |
|  | GDGGSTtGLSATPPASLPGSLTNVK | T396 |
|  | SSsAPNVHINTIEPVNIDDLIR | S365 |
|  | SNPKsPQKPIVR | S151 |
|  | DRsSSAPNVHINTIEPVNIDDLIR | S363 |
|  | **SASEPsLNR** | **S732** |
|  | sNPKsPQKPIVR | S147, S151 |
|  | **SAsEPsLNR** | **S729, S732** |
|  | sPGPQR | S419 |
|  | KSsSSSEDR | S428 |
| Sample H, cytosolic BRAF | IGSGsFGTVYK | S467 |
|  | RDsSDDWEIPDGQITVGQR | S446 |
|  | DQGFRGDGGSTTGLSAtPPASLPGSLTNVK | T401 |
|  | ALQKsPGPQR | S419 |
|  | GDGGSTtGLSATPPASLPGSLTNVK | T396 |
|  | SSsAPNVHINTIEPVNIDDLIR | S365 |
|  | SNPKsPQKPIVR | S151 |
|  | DRsSSAPNVHINTIEPVNIDDLIR | S363 |
|  | **SASEPsLNR** | **S732** |
|  | sNPKsPQKPIVR | S147, S151 |
|  | **SAsEPsLNR** | **S729, S732** |
|  | sPGPQR | S419 |
|  | KSsSSSEDR | S428 |
| Sample H, membrane recruited BRAF | GDGGStTGLSATPPASLPGSLTNVK | T395 |
|  | IHRSAsEPSLNR | S729 |
|  | ALQKsPGPQR | S419 |
|  | SNPKsPQKPIVR | S151 |
|  | GYLsPDLSK | S675 |
|  | **SAsEPsLNR** | **S729, S732** |
|  | SAsEPSLNR | S729 |
|  | KSsSSSEDR | S429 |
|  | IGsGSFGTVYK | S465 |
|  | RDsSDDWEIPDGQITVGQR | S446 |
|  | sPGPQR | S419 |
|  | sPQKPIVR | S151 |
|  | sPGPQRER | S419 |

**Supplementary Table S3.** ANOVA values of each figure before being subjected to a post-hoc test.

| Figure | Experiment | p-value |
| --- | --- | --- |
| 2 | BRAF CoIP (14-3-3 normalized to BRAF S732A-FLAG) | 0.00000634 |
| 2 | BRAF∆N-FLAG (14-3-3 normalized to BRAF∆N S732A-FLAG) | 0.000493693 |
| 3 | BRAF Homodimer (V5 normalized to BRAF S732A V5) | 2.01552E-05 |
| 3 | BRAF Homodimer (MEK activity) | 1.94E-07 |
| 3 | BRAF pT599 | 0.01464 |
| 3 | Phosphorylated MEK1 of kinase assay | 0.00892 |
| 4 | Dabrafenib BB, relative pMEK | 0.000962093 |
| 4 | Dabrafenib BB, relative pERK | 0.0316 |
| 5 | CRAF (14-3-3 normalized to A) | 0.00063419 |
| 5 | CRAF (pS338 normalized to A) | 0.04713 |
| 5 | CRAF Homodimer (MEK activity) | 0.63615 |
| 5 | BC Heterodimer (HA normalized to A) | 0.04649 |
| 5 | BC Heterodimer (MEK activity) | 6.60E-06 |
| 6 | BRAF full length with F (14-3-3 normalized to BRAF S732A-FLAG) | 0.01258 |
| 6 | BRAF pT599 normalized to A with F | 0.01922 |
| 6 | BRAF Homodimer (V5 normalized to BRAF S732E V5) | 0.0122 |
| 6 | BRAF Homodimer (MEK activity) | 3.23842E-06 |
| SI-Figure 1 | HELA WT A E F (14-3-3 RELATIVE TO A) | 0.02307 |
| SI-Figure 1 | HELA WT A E F (pT599 relative to A) | 0.00269 |
| SI-Figure 1 | HCT116 with WT A E F (Relative 14-3-3 to A) | 0.00483 |
| SI-Figure 1 | HCT116 with WT A E F (pT599 relative to A) | 0.04301 |
| SI-Figure 2 | BRAF S732F with Dabrafenib treatment (A vs F, Relative pMEK1/2) | 0.02316 |
| SI-Figure 2 | BRAF S732F with Dabrafenib treatment (A vs F, Relative pERK1/2) | 0.02023 |
| SI-Figure 2 | BRAF S732F with Dabrafenib treatment (E vs F, Relative pMEK1/2) | Reject null hypothesis: 0.74938 |
| SI-Figure 2 | BRAF S732F with Dabrafenib treatment (E vs F, Relative pERK1/2) | Reject null hypothesis: 0.61745 |
