# Supplementary material for: A novel phosphorylation site involved in dissociating RAF kinase from the scaffolding protein 14-3-3 and disrupting RAF dimerization
Source: J Biol Chem. 2023 Aug 23;299(10):105188. doi: 10.1016/j.jbc.2023.105188 (PMC10520314; doi:10.1016/j.jbc.2023.105188)
Supplement: Supporting figures [file mmc2.pdf]

Supporting Figures

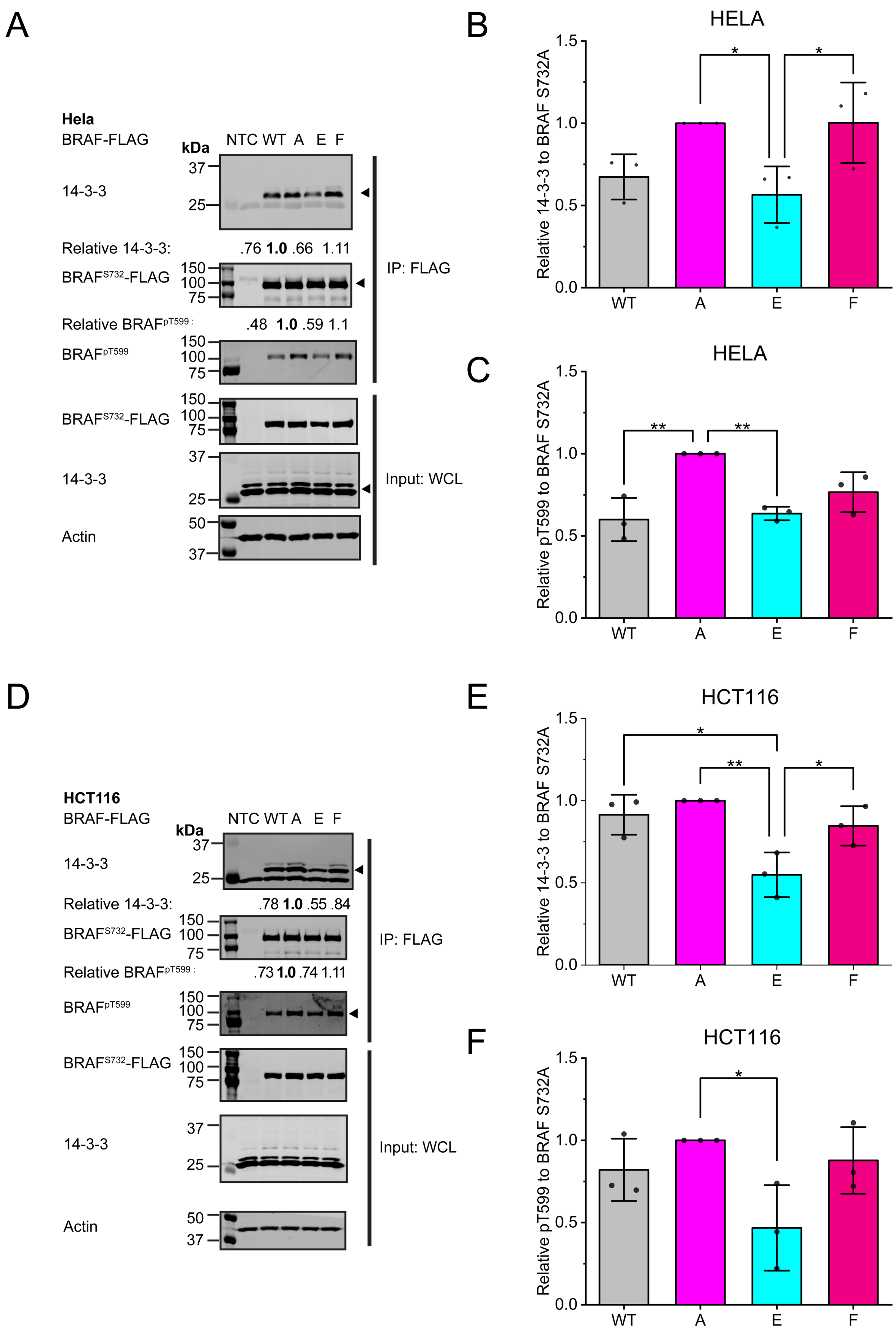
A
Hela
BRAF-FLAG
NTC WT A E F
kDa
14-3-3
37
25
Relative 14-3-3: .76 1.0 .66 1.11
BRAF^S732-FLAG
150
100
75
Relative BRAF^pT599: .48 1.0 .59 1.1
BRAF^pT599
IP: FLAG
BRAF^S732-FLAG
14-3-3
Actin
50
37
Input: WCL
B
HELA
Relative 14-3-3 to BRAF S732A
1.5
1.0
0.5
0.0
WT
A
E
F
*
C
HELA
Relative pT599 to BRAF S732A
**
D
HCT116
BRAF-FLAG
NTC WT A E F
kDa
14-3-3
Relative 14-3-3: .78 1.0 .55 .84
BRAF^S732-FLAG
Relative BRAF^pT599: .73 1.0 .74 1.11
BRAF^pT599
IP: FLAG
BRAF^S732-FLAG
14-3-3
Actin
Input: WCL
E
HCT116
Relative 14-3-3 to BRAF S732A
F
HCT116
Relative pT599 to BRAF S732A

Supporting Figure 1. Association of 14-3-3 and activation loop phosphorylation is not cell line specific with the phosphomimetic BRAF^{S732E} nor phosphodeficient BRAF^{S732A} models. A) The HELA cell line contains endogenous wild type, non-mutated MAPK pathway constituents, similar to the HEK293 cell line. Wild-type, phosphodeficient A, phosphomimetic E, and mutant phenylalanine BRAF^{S732}-FLAG were expressed in HELA cells and immunoprecipitated for the activation loop phosphorylation and coimmunoprecipitated the 14-3-3 association in the visualized representative immunoblot (n=3). B-C) Densitometry analysis of three biological replicates of relative 14-3-3 association (B) to BRAF^{S732A} and activation loop phosphorylation (C). Both 14-3-3 association and BRAF activation loop phosphorylation differ between the phosphomimetic and phosphodeficient models. Phosphodeficient A and mutant F associate with 14-3-3 differentially than phosphomimetic E. These analyses agree with the observations associated with the HEK293 experiments. D) The HCT116 cell line contains constitutively active upstream KRAS^{G13C}, which hyperactivates RAF kinase. Endogenous RAF kinases in this cell line are wild-type and non-mutated. BRAF WT and BRAF S→A/E/F mutants were expressed in HCT116 cells and immunoprecipitated for the activation loop phosphorylation status and coimmunoprecipitation of the 14-3-3 association (n=3). Representatives immunoblot is shown. E-F) Densitometry analysis of relative 14-3-3 and activation loop phosphorylation (F) to BRAF^{S732A} across three biological replicates. Despite hyperactive upstream activity, BRAF^{S732E} has the lowest 14-3-3 association and activation loop phosphorylation than the phosphodeficient A mutant. In an activating environment, BRAF^{S732F} is more similar to BRAF^{S732A}, based on no-significant differences observed with BRAF^{S732A}. All immunoblots contain their respective molecular weight markers where indicated. Black arrows indicate the correct band in blots with multiple bands. NTC is abbreviated for Non-Transfected Cells. Statistical significance was determined via one-way ANOVA, followed by the Tukey's HSD test. P-values are represented by: *P<0.05, **P<0.01, ***P<0.001.

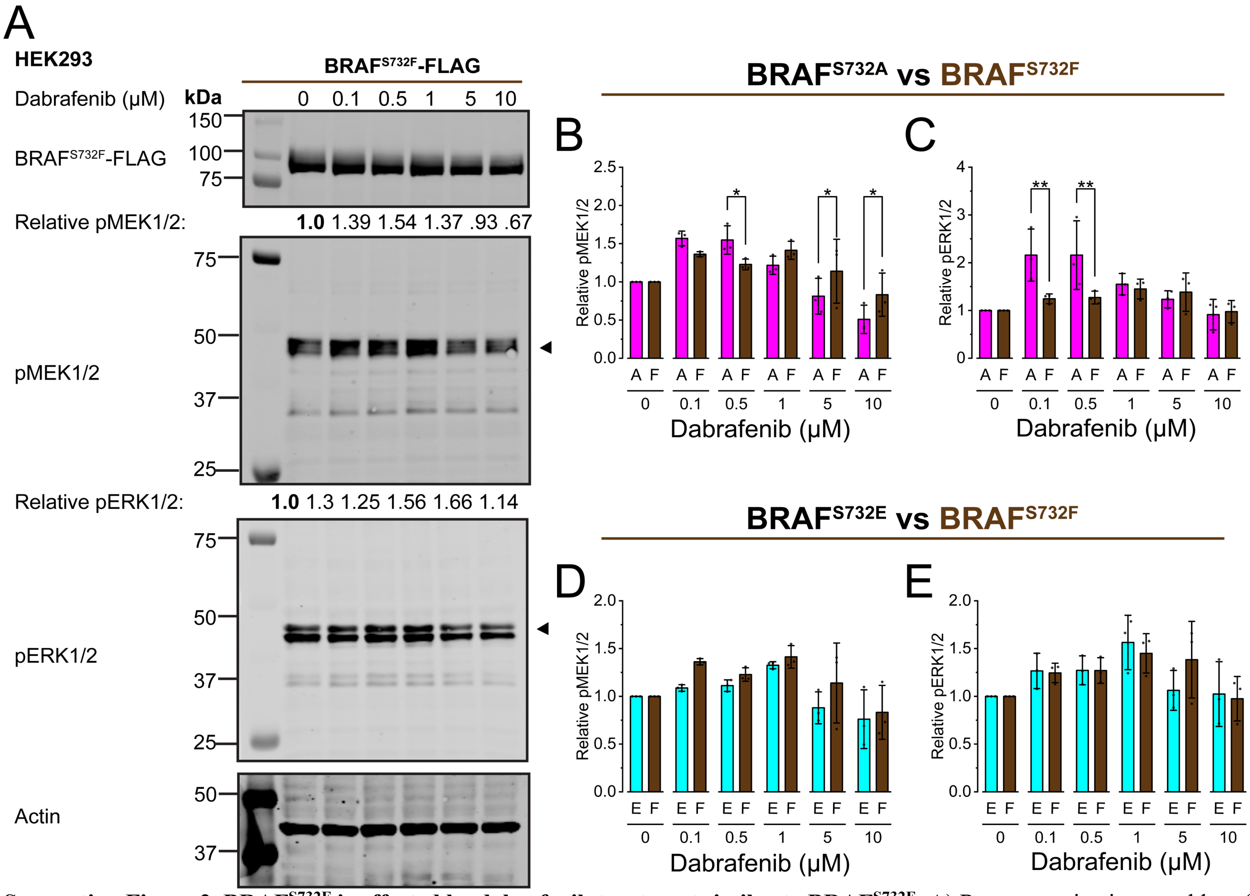

Supporting Figure 2. BRAF^{S732F} is affected by dabrafenib treatment similar to BRAF^{S732E}. A) Representative immunoblot of three biological replicates expressing BRAF^{S732F}-FLAG in HEK293 cells with 1-hour dabrafenib treatment (0-10 μM) (n=3). Phosphorylated MEK1/2 (pMEK1/2) and phosphorylated ERK1/2 (pERK1/2) are included as RAF-MEK1/2-ERK1/2 phosphorylated activity readouts of the MAPK pathway in response to dabrafenib treatment. The markers next to the immunoblots represent molecular weights in kDa. Relative phosphorylated MEK1/2 or ERK1/2 ratios are normalized to the 0 μM. The 0 μM accounts for the highest volume of DMSO. B-C) Densitometry analysis of three biological replicates for relative (B) pMEK1/2 and (C) pERK1/2 ratios comparing BRAF^{S732A} with BRAF^{S732F}. D-E) Densitometry analysis of three biological replicates for relative (D) pMEK1/2 and (E) pERK1/2 ratios comparing BRAF^{S732E} with BRAF^{S732F}. No significance is determined between the E and F mutants. All graph bars represent the mean± SD with individual data points per biological replicate. Statistical significance was determined via two-way ANOVA, followed by the post-hoc Holm-Bonferroni test for multiple comparisons. P-values are

represented by: *P<0.05, **P<0.01, ***P<0.001. Bar graphs are color coded in brown ($BRAF^{S732F}$), cyan ($BRAF^{S732E}$), and magenta ($BRAF^{S732A}$).
